# The Complete Genome and Physiological Analysis of the Eurythermal Firmicute *Exiguobacterium chiriqhucha* Strain RW2 Isolated From a Freshwater Microbialite, Widely Adaptable to Broad Thermal, pH, and Salinity Ranges

**DOI:** 10.3389/fmicb.2018.03189

**Published:** 2019-01-08

**Authors:** Richard Allen White, Sarah A. Soles, Greg Gavelis, Emma Gosselin, Greg F. Slater, Darlene S. S. Lim, Brian Leander, Curtis A. Suttle

**Affiliations:** ^1^Department of Microbiology and Immunology, University of British Columbia, Vancouver, BC, Canada; ^2^School of Geography and Earth Sciences, McMaster University, Hamilton, ON, Canada; ^3^Department of Zoology, University of British Columbia, Vancouver, BC, Canada; ^4^Department of Earth, Ocean and Atmospheric Sciences, University of British Columbia, Vancouver, BC, Canada; ^5^Bay Area Environmental Institute, Petaluma, CA, United States; ^6^NASA Ames Research Center, Moffett Field, CA, United States; ^7^Department of Botany, University of British Columbia, Vancouver, BC, Canada; ^8^Institute for the Oceans and Fisheries, University of British Columbia, Vancouver, BC, Canada

**Keywords:** exiguobacterium, microbialite, cosmopolitan, heavy metals, metabolic potential

## Abstract

Members of the genus *Exiguobacterium* are found in diverse environments from marine, freshwaters, permafrost to hot springs. *Exiguobacterium* can grow in a wide range of temperature, pH, salinity, and heavy-metal concentrations. We characterized *Exiguobacterium chiriqhucha* strain RW2 isolated from a permanently cold freshwater microbialite in Pavilion Lake, British Columbia using metabolic assays, genomics, comparative genomics, phylogenetics, and fatty acid composition. Strain RW2 has the most extensive growth range for temperature (4–50°C) and pH (5–11) of known *Exiguobacterium* isolates. Strain RW2 genome predicts pathways for wide differential thermal, cold and osmotic stress using cold and heat shock cascades (e.g., *csp* and *dnaK*), choline and betaine uptake/biosynthesis (e.g., *opu* and *proU*), antiporters (e.g., *arcD* and *nhaC* Na^+^/K^+^), membrane fatty acid unsaturation and saturation. Here, we provide the first complete genome from *Exiguobacterium chiriqhucha* strain RW2, which was isolated from a freshwater microbialite. Its genome consists of a single 3,019,018 bp circular chromosome encoding over 3,000 predicted proteins, with a GC% content of 52.1%, and no plasmids. In addition to growing at a wide range of temperatures and salinities, our findings indicate that RW2 is resistant to sulfisoxazole and has the genomic potential for detoxification of heavy metals (via mercuric reductases, arsenic resistance pumps, chromate transporters, and cadmium-cobalt-zinc resistance genes), which may contribute to the metabolic potential of Pavilion Lake microbialites. Strain RW2 could also contribute to microbialite formation, as it is a robust biofilm former and encodes genes involved in the deamination of amino acids to ammonia (i.e., L-asparaginase/urease), which could potentially boost carbonate precipitation by lowering the local pH and increasing alkalinity. We also used comparative genomic analysis to predict the pathway for orange pigmentation that is conserved across the entire *Exiguobacterium* genus, specifically, a C_30_ carotenoid biosynthesis pathway is predicted to yield diaponeurosporene-4-oic acid as its final product. Carotenoids have been found to protect against ultraviolet radiation by quenching reactive oxygen, releasing excessive light energy, radical scavenging, and sunscreening. Together these results provide further insight into the potential of *Exiguobacterium* to exploit a wide range of environmental conditions, its potential roles in ecosystems (e.g., microbialites/microbial mats), and a blueprint model for diverse metabolic processes.

## Introduction

Microbialite fossils (i.e., the ancient stromatolites) represent the oldest evidence for life on the Earth (Nutman et al., 2016). Microbialites consist of a specialized microbial mat that lithifies carbonates into two main structural types, (1) thrombolites; composed of unlaminated clots, or (2) stromatolites; defined by laminated layers (Burne and Moore, 1987; Perry et al., 2007). Microbialites represent natural analogs for the early microbial ecosystems, which allow for testing hypotheses around the basic principles of microbial ecology including questions regarding community composition (Wong et al., 2015, 2017), community assembly (Havemann and Foster, 2008), functional traits and diversity (Breitbart et al., 2009; White et al., 2015, 2016; Ruvindy et al., 2016; Wong et al., 2018) and discovery of novel taxa (Burns et al., 2012; White et al., 2018b).

Our model microbialite ecosystem is Pavilion Lake a cold, oligotrophic ecosystem in southeastern British Columbia, Canada with dimictic circumneutral waters (median pH 8.3; mean calcium carbonate, 182 mg L^-1^), (White et al., 2016). Characterization of the limnology of Pavilion Lake is described in detail Lim et al. (2009). Resident microbialites are calcium carbonate-based thrombolites whose morphological features vary with depth (White et al., 2016), though all have thin (∼5 mm) microbial mats dominated by cyanobacteria.

While both heterotrophs and photoautotrophs (i.e., cyanobacteria) have been described and isolated from a range of microbial mats including microbialites, little work has been done on pigmented heterotrophic bacteria within microbialites. *Firmicutes* have been identified in the pigmented layers in microbial mats (Bottos et al., 2008; Lionard et al., 2012), and it is thought that carotenoids are responsible for their characteristic coloration (Nübel et al., 1999; Mueller et al., 2005; Klassen, 2010; Fisher et al., 2019). Fundamental questions about heterotrophic non-phototrophic arise which include (1) what the function of pigments in these microbialite associated heterotrophic bacteria is? and (2) what is the role of pigmented heterotrophs in ecosystem functioning within microbialites? To address these questions, we enriched and isolated >100 pigmented bacteria from Pavilion Lake freshwater microbialites, and grew them in the dark to select for heterotrophic or mixotrophic strains.

**FIGURE 1:**
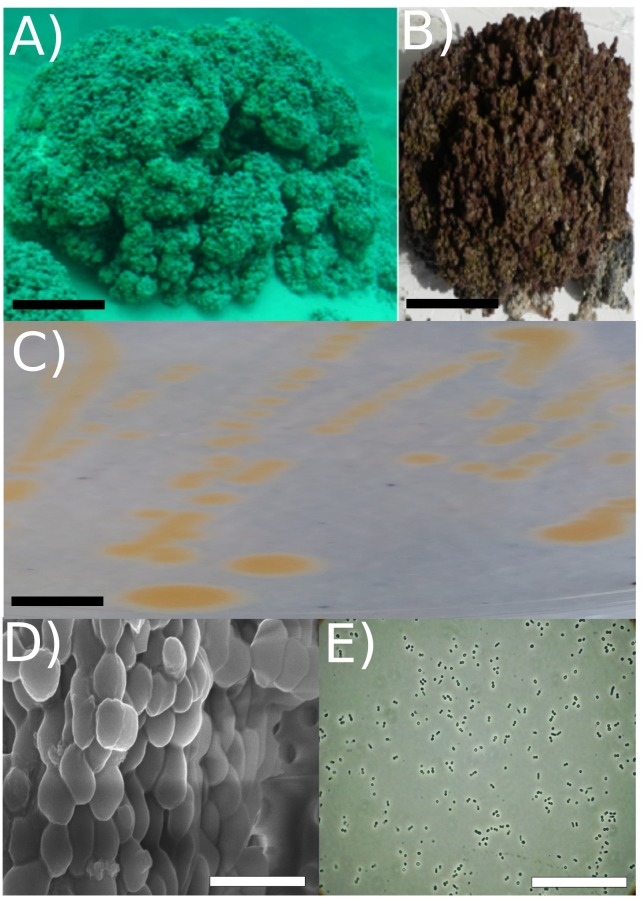
Strain RW2 isolation environment, culture plate illustration, and microscopy. **(A)** Picture of Pavilion Lake microbialite (i.e., thrombolite) taken underwater (20 m depth sample, scale bar: ∼1 m). **(B)** Pavilion Lake microbialite (i.e., thrombolite) taken at sample station (20 m depth sample, scale bar: ∼15 cm). **(C)** Culture plate (∼24 h growth, the black scale bar ∼10 mm). **(D)** Light microscopy (∼24 h growth, scale bar: ∼80 μm); **(E)** SEM (∼72 h growth, scale bar: ∼5 μm). All microscopy (i.e., SEM/light) and culture plate photo **(C–E)** were completed at strain RW2 standard growth parameters on solidified M-agar at 30°C, pH 7 and concentration 1% (w/v) NaCl. Panels **(A,B)** adapted from White et al. (2018a).

Among these pigmented heterotrophic enrichments included *Exiguobacterium* “strain RW2,” a Gram-positive member of the *Firmicutes.* Strain RW2 was isolated and enriched from a thrombolite at 20 m depth, where the water temperature remains around 4–10°C throughout the year (Lim et al., 2009) (Figures 1A,B). Bacteria at this depth should be adapted to cold temperature, low phosphorus, and alkaline conditions. Recently, we have described another heterotrophic pigmented isolate *Agrococcus pavilionensis* strain RW1 (White et al., 2013b, 2018a), that was co-isolated with strain RW2 from the same microbialite and enrichment.

Members of this genus have a cosmopolitan distribution due to their highly adaptable physiology. Isolates of *Exiguobacterium* represent two major clades, clade I, which is comprised of cold-adapted strains including *E. sibiricum* 255-15^T^ (Rodrigues et al., 2008), and clade II, whose members range from temperate (e.g., *E. aurantiacum* DSM6208^T^) to hot environments (e.g., *E.* sp. AT1b) (Vishnivetskaya et al., 2009; Gutiérrez-Preciado et al., 2017), and include strain RW2. Due to the global distribution of *Exiguobacterium*, microbialites and/microbial mats appear to be another environment where they can adapt and colonize. Two *Exiguobacterium* strains have been directly isolated from microbialites: the present strain RW2 (formally *pavilionensis*) isolated from cold freshwater thrombolites (White et al., 2013a; Gutiérrez-Preciado et al., 2017), and strain S17, which was isolated from a warm (i.e., ∼20–24°C) freshwater stromatolite in Lake Socompa (Ordoñez et al., 2013, 2015). Based on average nucleotide identity (ANI), RW2 appears to be the same species as two other strains; strain N139 and GIC31 (formally *pavilionensis*, Gutiérrez-Preciado et al., 2017), and it represents the type strain. Strain N139 was isolated from the water column of Leguna Negra which contains microbialites/microbial mats with extreme temperature variation ranging from -5 to 42°C (Gomez-Javier et al., 2018; Mlewski et al., 2018). Remarkably, the third strain—GIC31 was isolated from glacial ice (Vishnivetskaya et al., 2009), potentially from a biofilm growing on top of the ice, but this is unknown. Another question arises (3) are strains RW2, N139, and GIC31 the same species? We use comparative genomics to determine the species placement of these three strains (i.e., RW2, N139, GIC31).

At the time of this writing, the genus *Exiguobacterium* has sixteen named species (Table 1), which were isolated from diverse environments including permafrost (Rodrigues et al., 2006), deep-sea vents (Crapart et al., 2007) and hot springs (Vishnivetskaya et al., 2009, 2011). These widespread bacteria can tolerate of a wide range of pH (5–11), salinity (NaCl: 0–16%) and temperature (-12°C to 55°C) (White et al., 2013a) (Table 1). The first described member of the genus, *E. aurantiacum* DSM6208^T^, was isolated from a potato processing plant (Collins et al., 1983). Consequently, isolates of *Exiguobacterium* spp. have been used as model organisms for understanding thermal adaptation to cold (i.e., -5°C) (Rodrigues et al., 2008; Vishnivetskaya et al., 2009), hot temperatures (i.e., 55°C) and heavy metal stress (Ordoñez et al., 2013, 2015). Currently, 60 genomes of *Exiguobacterium* are listed on NCBI, 17 of which are complete (Supplementary Table S1). However, no complete genomes are available from microbialites, from which only a draft-genome is available (White et al., 2013a).

**Table 1:** Names and isolation location of *Exiguobacterium* species.

Species Name	Environment of Isolation	Reference	Isolation Notes
**ICSP recognized species**
*E. acetylicum*	Creamery waste	Farrow et al., 1994	Levine and Soppeland, 1926 classified “*Flavobacterium acetylicum*” renamed “*Brevibacterium acetylicum*” then reclassified by Farrow et al. (1994)
*E. antarcticum*	Microbial mat/biofilm	Fruhling et al., 2002; Carneiro et al., 2012	Lake Fryxell mat in Antarctica
*E. aestuarii*	Marine tidal flat	Kim et al., 2005	Daepo beach in yellow sea near Mokpo City, Korea,
*E. alkaliphilum*	Wastewater drained sludge	Kulshreshtha et al., 2013	Beverage industry facility New Delhi, India
*E. aquaticum*	Freshwater lake	Raichand et al., 2012	Tikkar Tal Lake, Haryana, India
*E. aurantiacum*	Potato processing plant	Collins et al., 1983	**Type species for the genus**
*E. himgiriensis*	Soil	Singh et al., 2013	Lahaul-Spiti valley, Indian Himalayas
*E. indicum*	Glacial melt water	Chaturvedi and Shivaji, 2006	Hamta glacier (4270 m above sea level), Himalayans
*E. martemiae*	Brine shrimp cysts	Lopez-Cortes et al., 2006	*Artemia franciscana* cysts
*E. mexicanum*	Brine shrimp cysts	Lopez-Cortes et al., 2006	*Artemia franciscana* cysts
*E. oxidotolerans*	Fish processing plant	Yumoto et al., 2004	Hokkaido, Japan
*E. profundum*	Hydrothermal Vent	Crapart et al., 2007	Northeast Pacific rise at ∼2600 m
*E. sibiricum*	Permafrost	Rodrigues et al., 2006	Siberian permafrost (3 Mya old)
*E. soli*	Glacial moraine	Chaturvedi et al., 2008	McMurdo dry valleys, Antarctica
*E. undae*	Garden pond water	Fruhling et al., 2002	Wolfenbüttel, Lower Saxon Germany
**Published strains discussed**
Strain RW2	Thrombolithic microbialite	White et al., 2013a	Pavilion lake 20 m microbialites
Strain S17	Stromatolithic microbialite	Ordoñez et al., 2013	Polyextremophile strain tolerate heavy metals (As)
Strain AT1b	Alkaline hot spring	Vishnivetskaya et al., 2011	Mammoth Terrace hot spring YNP, Wyoming, United States
Strain N139	Hypersaline lagoon	Gutiérrez-Preciado et al., 2017	Polyextremophile strain tolerate heavy metals (As)
Strain GIC31	Glacier ice	Vishnivetskaya et al., 2011, 2014	Greenland ice shelf


*Official species names are recognized by the International Committee on Systematics of Prokaryotes (ICSP) and have been published in the International Journal of Systematic and Evolutionary Microbiology or another journal recognized by the ICSP. Isolates published in NCBI as genome announcements or other that are unnamed, not classically described, or not recognized by the ICSP are listed as *Exiguobacterium* sp.*

Here, we present the first such complete genome of *Exiguobacterium* isolated from modern microbialites. We also characterize strain RW2 using standard bacteriological and physiological testing, comparative genomics, and phylogenetics (i.e., 16S rRNA gene phylogeny) to describe its placement in the genus, and its broad adaptation to ranges of salinity, pH, and temperature. Lastly, from a genomic standpoint, we assess the potential of strain RW2 to contribute important metabolic functions to the microbialite community in Pavilion Lake.

## Materials and Methods

### Isolation, Growth Conditions, Biochemical and Antibiotic Susceptibility Tests

Strain RW2 was isolated by plating 0.5 g of homogenized thrombolytic microbialite, collected from 20 m depth in Pavilion Lake, British Columbia (50.86°N, 121.74°W), onto M-agar medium [0.5% (w/v) tryptone, 0.25% (w/v) yeast extract, 1% (w/v) NaCl, 1.5% (w/v) agar, pH 7], followed by incubation at 30°C for 3 days in the dark (White et al., 2013a) (Figure 1C). M-agar plates (1% NaCl, pH 7, 1.5% agar w/v) were also used for culture maintenance (at 30°C) and for assessing growth under the following conditions. Growth effects of temperature were measured at 4, 5, 11, 16, 18, 20, 25, 30, 37, 42, 45, and 50–55°C. At temperatures greater than 45°C, we used thicker M-agar plates at 4% (w/v) agar, to avoid dehydrating the plates. Growth effects of pH were measured at 4, 5, 6, 6.5, 7, 7.5, 8, 10, 10.5, 11, and 12 pH. Lastly, growth effects of salt were measured with 0, 1, 3, 6, 9, 12, 13, 16% added NaCl. Note that these percentages reflect that of added NaCl and not salinity, as tryptone and yeast extracts contain preexisting salts. Each condition was imposed for 72 h on triplicate plates. Standard colony forming unit (CFU) evaluation was used in that growth occurred only when >100 CFUs occurred on plates. All grow evaluations on plates were confirmed in liquid culture followed by optical density measurement.

We also assessed photoautotrophic and photoheterotrophic growth on MM9 minimal medium [20% glucose, 0.5% (w/v) tryptone, 0.25% (w/v) yeast extract], and on RCV medium (Beatty and Gest, 1981) in liquid medium and agar plates (1.5% agar w/v) grown both anaerobically, microaerophilically and aerobically with and without ambient sunlight on 8 h light and 16 dark cycle. No photoautotrophy or photoheterotrophy was observed.

Strain characteristics, including colony and cell morphologies, were determined by standard methods (Murray et al., 1994). Oxidase tests and biochemical enzyme assays and carbohydrate use were conducted in triplicate using API20E (BioMérieux) test strips following manufacturers instructions. Single isolated colonies from M-agar plates were washed via pelleting at 3,250 × *g* for 10 min three times in sterile distilled water then inoculated to API20E (BioMérieux) test strips, which includes a motility assay. Antibiotic susceptibility was determined by the Kirby-Bauer method using antibiotic disks on M-agar plates in triplicate (Collee et al., 1996).

Triplicate experiments in liquid culture were used to induce biofilm formation and measure timing (M-broth 30°C at pH 7 and 1% NaCl w/v) at low shaking (100 rpms) and without shaking. A positive biofilm in strain RW2 was a top film appeared over medium within the flask.

### Phospholipid Fatty Acid Analysis (PLFA)

Phospholipid fatty acids (PLFAs) were extracted from cultures grown in triplicate M-agar plates for 72 h at multiple conditions for temperatures (4, 18, 30, 50°C at pH 7 and 1% NaCl, 1.5% agar w/v, 4% agar w/v at 50°C), pH (5, 7, 11 at 30°C at 1% NaCl w/v in 1.5% agar w/v) and added NaCl (5, 7, 11 at 30°C at 1% NaCl w/v in 1.5% agar w/v). All PLFA results were confirmed in liquid medium. Cultures were transferred into pre-combusted vials for an overnight solvent extraction in a 1:2:0.8 ratio of dichloromethane (DMC), methanol (MeOH) and phosphate-buffered saline (PBS) [137 mM NaCl, 2.7 mM KCL, 10 mM Na_2_HPO_4_ 2H_2_O, 2 mM KH_2_PO_4_, pH 7.4] (Bligh and Dyer, 1959). The extract was filtered through a separatory funnel where DMC and water were added to achieve a mixture of MeOH:DMC:water of 1:1:0.9 (Bligh and Dyer, 1959). The lower organic phase was removed and purified into polar, neutral, and non-polar fractions using liquid chromatography through silica gel. Phospholipids present in the polar fraction were subjected to mild alkaline methanolysis to produce fatty acid methyl esters (FAMEs) (Guckert et al., 1985). FAMEs were separated, identified, and quantified using gas chromatography-mass spectrometry (GC/MS) (Agilent Technologies Inc., Santa Clara, CA, United States) with a DB-5MS capillary column (30 m × 0.32 mm I.D. × 0.25 μm film thickness) under a temperature regime of 50°C (1 min), 20°C min^-1^ to 130°C, 4°C min^-1^ to 160°C, and 8°C min^-1^ to 300°C (5 min). PLFAs were identified by retention time and mass spectra relative to those of reference standards (Bacterial Acid Methyl Ester Mix, Matreya Inc., Pleasant Gap, PA, United States; and Supelco 37 Component FAME Mix, Sigma-Aldrich Co., Bellefonte, PA, United States). A modified picolinyl ester derivatization was used to determine the branching point in unknown compounds (Dowd, 1998; Destaillats and Angers, 2002). Dimethyl disulfide derivatives were prepared to determine the double bond position in unsaturated fatty acids (Nichols et al., 1986).

### Light and Scanning Electron Microscopy (SEM)

Exponentially growing cells were harvested at 26 h after being transferred to liquid M-medium and were viewed by light microscopy under oil immersion at 100×. Flagella straining was completed as per Kodaka et al. (1982) then viewed by light microscopy after 26 h of growth. For SEM, a culture in stationary phase ∼72 h after being transferred to liquid M-medium was pelleted at 3,250 × *g* for 10 min, then the M medium was exchanged and cells were fixed in 2.5% glutaraldehyde in PBS (137 mM NaCl, 2.7 mM KCL, 10 mM Na_2_HPO_4_ 2H_2_O, 2 mM KH_2_PO_4_, pH 7.4) solution for 30 min on ice. The fixed culture was filtered onto a 0.2 μm pore-size Supor polycarbonate membrane (Pall Port Washington, NY, United States). Cells on the filter were washed with PBS and post-fixed in 1% OsO_4_ for 1 h. The cells and filter were passed through a graded ethanol series (25, 50, 70, 95, 100%) at 10 min intervals, and in 100% ethanol were critical-point dried with CO_2_. A sputter coater applied 5 nm of gold/palladium alloy onto the cells before imaging by SEM using a Hitachi S4700 microscope.

### Phylogenetic Analysis of 16S rRNA Gene Sequences

Thirty-two reference sequences were downloaded from NCBI representing all named typed (^T^) strains of genus *Exiguobacterium*, with three typed strains of *Bacillus* spp. as outgroups. The whole-genome assembled 16S rRNA gene sequence was used for phylogenetic analysis. Alignments of 16S rRNA gene sequences were completed in MAFFT (v. 7.310) using the options (–localpair –maxinterate 1000) which is iterative refinement method incorporating local pairwise alignment information (L-INS-i) providing the most accurate alignment (Katoh et al., 2002). A maximum likelihood phylogenetic tree was constructed using IQ-TREE (v. 1.6.1) (Hoang et al., 2017) with a total of 1,000 bootstrap replicates using UFBoot2 (Trifinopoulos et al., 2016), and visualized with SeaView (v. 4) (Gouy et al., 2010). Strain RW2 16S rRNA gene sequence and alignments are available on github.com/strain_RW2.

### Whole Genome Assembly and Annotation

DNA extraction, Illumina library preparation and sequencing, data cleaning, phiX spike-in removal, and draft genome assembly are described by White et al. (2013a). The genome was closed into a single circular contig (i.e., chromosome) without plasmids using comparative genome ordering, alignment, mapping, and manual editing. For comparison, scaffolding and contig ordering of the genome of strain AT1b was compared to strain RW2 which was downloaded from NCBI. Strain RW2 contigs were ordered and aligned in progressiveMauve to strain AT1b to visualize genome order (Darling et al., 2010; Vishnivetskaya et al., 2011). The remaining gaps between contigs were closed by recursive alignments using Mauve. The ordered contigs with overlaps were merged into a single circular contig using the EMBOSS union script (Rice et al., 2000). To estimate sequencing depth represented in the assembly for coverage estimation, reads used in the assembly were mapped back to the final circular genome using Bowtie2 with the very sensitive local option (Langmead and Salzberg, 2012). The Bowtie2 read mapping output file (Sam file) was visually inspected by the Tablet program (Milne et al., 2013). The contigs that were screened for overlaps and read mapping depth (>10×) were then merged manually, based on the reference genome of AT1b.

Annotation was conducted on the RAST annotation server using the Glimmer-3 option and standard RAST against the FIGfam database release-70 (Aziz et al., 2008). Metabolic pathways were predicted in strain RW2 using MetaPathways which uses pathway tools (Paley and Karp, 2006; Konwar et al., 2013; Caspi et al., 2014), against MetaCyc/BioCyc databases via a built-in LAST (Local Alignment Search Tool) (Kiełbasa et al., 2011) for homology matches of ≥180 bp ORFs (protein-coding open reading frames) and ≥50 alignment score. A metabolic model for strain RW2 was predicted using ModelSEED (Devoid et al., 2013). The Prokaryotic Genome Annotation Pipeline (PGAP) at NCBI was also used to compare to RAST annotation for strain RW2 (Tatusova et al., 2016). We manually curated the annotations produced by RAST and NCBI (PGAP)—retaining only annotations that were a consensus between both programs.

### Comparative Genomic Analysis

All genomes available for *Exiguobacterium* were uploaded to RAST, for synteny, AAI via RAST annotations, and metabolic model comparisons in modelSEED. Metapathways was also used to predict pathways across the genomes using BioCyc/MetaCyc. To display genomic features a circular genome plot of strain RW2 was constructed using Cgviewer server^1^ (Grant and Stothard, 2008, version V 1.0, date accessed Jan 1st, 2018). Genomes of strains RW2, S17, GIC31, and N139 were compared in CGViewer using tBLASTx (e-value of 1e^-5^, with 70% identity cutoff, and minimum 50 bp overlap) and then displayed in the CGviewer genome plot server (Grant and Stothard, 2008). Synteny plots were completed in the RAST server module using a BLAST-based dot plot format. Average amino-acid identity (AAI) and Reciprocal Orthology Score Average (ROSA) via functional gene similarities from RAST server annotations were calculated using with a web-based tool^2^ (Krebs et al., 2013, date accessed January 1st, 2018).

For species classification, average nucleotide identity (ANI), AAI (non-RAST based), and digital DNA–DNA hybridization were used to compare strains (i.e., RW2, N139, and GIC31). ANI/AAI was completed using web server from Kostas lab^3^ with default parameters based on the enveomics software package collections (Rodriguez-R and Konstantinidis, 2016, date accessed August 1st, 2018). Digital DNA–DNA hybridization for species determination was completed using GGDC (v. 2.1) web server (Meier-Kolthoff et al., 2013, 2014, date accessed August 1st, 2018).

### Metagenomic Read Recruitment

The presence of strain RW2 within Pavilion Lake sediments, microbialites, and water column was previously described in a metagenomic study by White et al. (2016). We compared our genomic data to their published metagenomic data, by loading their predicted protein dataset onto MG-RAST (Meyer et al., 2008), and using RefSeq (protein level) annotations (using BLAT parameters, with matches of ≥60% similarity cutoff, ≥15 bp overlap, and minimum E-value of 10^-5^) to search for signatures of strain RW2 in their Pavilion Lake metagenomes. Reads assigned to *Exiguobacterium* from the metagenome were mapped to the strain RW2 using Bowtie2 with the very sensitive local option (Langmead and Salzberg, 2012). These results were subject to ANOVA statistical testing using the Statistical Analysis of Metagenomic Profiles package (STAMP) (Parks and Beiko, 2010). The program FR-hit was then used to assign metagenomic read recruitment of the 7.5 million Metagenomic sequences (250 bp paired-end MiSeq reads) to *Exiguobacterium* genomes from strain RW2, S17, and AT1b, using default parameters with a minimum identity > 70% and an E-value > 1e^-5^ (Niu et al., 2011). The recruitments were then visualized with the R library ggplot2 (Wickham, 2016).

### Data Availability

*S*train RW2 is listed at NCBI under bioproject accession PRJNA208114 and biosample accession SAMN02471612. The Ray assembly for the draft genome is listed under DDBJ/EMBL/GenBank under the accession number ATCL00000000, and RefSeq under NZ_ATCL00000000.1, as well as NCBI under GCA_000416965.1. Lastly, the complete genome, scripts, and supplementary data are posted on github.com/raw937/Strain_RW2/.

## Results

### Classification of RW2 Related Strains Using Phylogenetics and Whole-Genome Analysis

Using 16S rRNA gene-based phylogeny, whole-genome ANI/AAI and digital DNA–DNA hybridization we will determine whether RW2, GIC31, and N139 are the same species. Currently, strains GIC31 and N139 are missing data required for classical bacteriological species determination via the IJSEM which include experimental fatty acid analysis, quinone characterization, peptidoglycan structure analysis, and DNA–DNA hybridization assays. However, a recent proposal from IJSEM suggests that genome-based ANI/AAI and 16S rRNA gene-based phylogeny can replace the experimental DNA–DNA hybridization and be used for naming/typing strains in the future (Chun et al., 2018).

**FIGURE 2:**
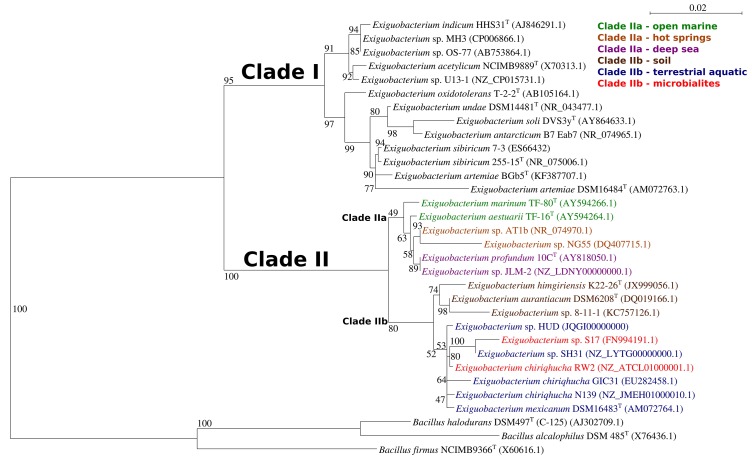
Maximum-Likelihood tree based on 16S sequences showing the phylogenetic relationship among different isolates of the genus *Exiguobacterium.* Bootstrap values greater than 50% are given at nodes based on 1000 replications. Bar represents a nucleotide substitution rate per 1000 nucleotides. The named, typed strains are labeled (^T^), other strains are published as in NCBI or as genome announcements.

We generated a maximum-likelihood phylogeny of 16S rRNA gene sequences from all fourteen typed strains of *Exiguobacterium* spp., fifteen sequences representing strains with complete genomes, and three typed strains of *Bacillus* spp. as outgroups. This positioned strain RW2 in clade II, along with *E*. sp. AT1b and *E. aurantiacum* DSM6208^T^ (Figure 2). *E. aurantiacum* DSM6208^T^ represents the first isolate of *Exiguobacterium*, from potato effluent (which contains soil) (Collins et al., 1983). Within clade II are two subclades; clade IIa and clade IIb, together containing isolates from a wide range of environments (Figure 2). Clade IIa stem from hot springs and both shallow and deep-sea marine ecosystems, whereas isolates of clade IIb stem terrestrial habitats including soil, microbialites (including strain RW2), and freshwater ecosystems (Figure 2). Clade IIb is well-supported, suggesting that it represents a true radiation into terrestrial habitats including lakes (i.e., strains N139, SH31, S17, RW2), glacial ice (i.e., strain GIC31), microbialites (i.e., strains S17, RW2), canals (i.e., strain HUD), and even the microbiomes of brine shrimp, which dwell in hypersaline vernal pools (i.e., strain DSM16483^T^) and canals (i.e., strain HUD) (Figure 2). However, the subclade that contains strains RW2, N139, GIC31, and HUD is not well resolved (Figure 2). Strain HUD is currently classified by NCBI as a strain of *E. mexicanum*, but this is challenged by our 16S rRNA gene phylogeny, given that it does not branch with the type strain of *E. mexicanum* (strain DSM16483) (Figure 2). The genome of the type strain of *E. mexicanum* DSM16483^T^ is currently not available to compare to strain HUD or other strains related to strain RW2. There is high bootstrap support for a clade containing strain RW2 (as the basal member) along with strains SH31 and S17. By contrast, strains HUD, GIC31, and N139 branch earlier than this clade, where the tree topology is poorly resolved (i.e., the branches are polytomic) (Figure 2). The 16S rRNA gene of RW2 is 99% similar to GIC31 and N139 based on BLASTN, whereas N139 is 100% similar to GIC31. More representative 16S rRNA gene sequences are needed to resolve the polytomy within clade IIb (Figure 2).

**FIGURE 3:**
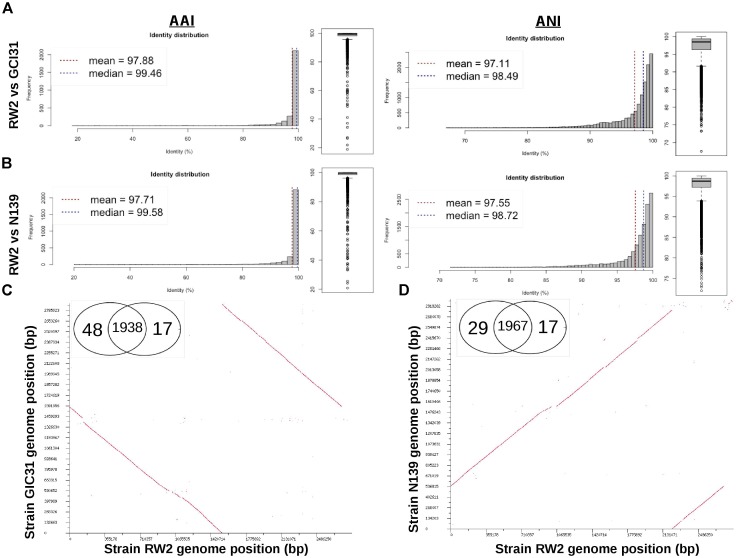
Whole genome comparison of strain RW2, N139, and GIC31 for average amino acid identity (AAI), average nucleotide identity (ANI), genome synteny and Venn diagrams of RAST SEED functions. **(A)** Strain RW2 vs. GIC31 for AAI/ANI using enveomics. **(B)** Strain RW2 vs. N139 for AAI/ANI using enveomics. **(C)** Strain RW2 vs. GIC31 - BLAST dot-plots for genome synteny and Venn diagram. **(D)** Strain RW2 vs. N139 - BLAST dot-plots for genome synteny and Venn diagram. Genomes are listed in megabase pairs (Mb). Red dots are positive blast hits based on the RAST genome comparison module. Venn diagrams are listed by strain name based on RAST functional annotations (SEED/FigFams).

**FIGURE 4:**
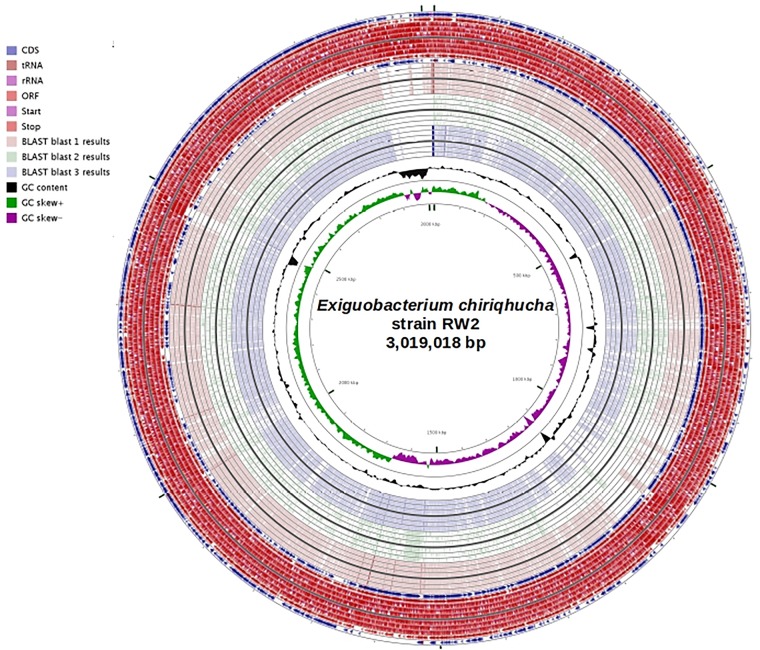
Genome plot of strain RW2 (bp) using CGviewer. Genome key (left corner): starts with the innermost ring which is a genome ruler followed by GC skew (purple/green) and ends with two outer rings which contain Protein coding ORFs (blue), tRNAs and rRNAs (red). tBLASTx was used compare RW2 with an e-value 1e^-5^, alignment cutoff – 50 bp, identity cutoff – 70%. Blast 1 is strain N139 vs. RW2, Blast 2 is strain S17 vs. RW2, and Blast 3 is strain GIC31 vs. RW2.

Based on 16S rRNA gene sequence alone it is unclear whether strains RW2, GIC31, and N139 belong to the same species. Whole-genome analysis is needed to resolve this issue. Strain RW2, N139, and GIC31 have >97% similarity both on the nucleotide (ANI) and protein (AAI) level (Figures 3A,B). Based on standards established in *E. coli*, >95% similarity between the 16S rRNA gene sequence of two isolates is used as a cutoff for conspecificity (Rodriguez-R and Konstantinidis, 2016). Furthermore, our genomic analysis suggests that gene content and synteny are highly conserved across RW2, GIC31, and N139 (Figures 3C,D). Based on RAST functional assignment, all three strains have >1900 shared functions (97% shared) with <50 functions (3% distinct) that are unique to each strain (Figures 3C,D). Likewise inter-strain tBLASTx used in the CGViewer estimated >97% homology for GIC31 vs. RW2, >97% N139 vs. RW2 and 87% for S17 vs. RW2 (Figure 4). Lastly, Digital DNA–DNA hybridization (dDDH) estimated that RW2, GIC31, and N139 are the same species with >80% probability based on logistic regression, with >70% being the cut-off for species determination (Table 2). Both N139 and GIC31 contain plasmids, though we found no evidence of plasmids in strain RW2 (Table 2). The tBLASTx found (>97%) homology for GIC31 vs. RW2, N139 vs. RW2 and 87% for S17 vs. RW2 (Figure 4).

**Table 2:** Digital DNA–DNA hybridization (dDDH) using GGDC 2.1 for strain RW2, GIC31, N139.

	Value	Range	Notes
**Strain RW2 vs. N139**			
*DDH (identities/HSP length – formula 2)*
*Distance:*	0.0256		
*DDH estimate (GLM-based):*	78.10%	75.2–80.8%	
*Probability that DDH > 70% (i.e., same species):*	89.02%		(via logistic regression)
*Probability that DDH > 79% (i.e., same subspecies):*	44.21%		(via logistic regression)
**Strain RW2 vs. GIC31**			
*DDH (identities / HSP length – formula 2)*			
*Distance:*	0.0302		
*DDH estimate (GLM-based):*	74.50%	71.4–77.3%	
*Probability that DDH > 70% (i.e., same species):*	85.09%		(via logistic regression)
*Probability that DDH > 79% (i.e., same subspecies):*	37.15%		(via logistic regression)


*Cut-offs for same species with >80% probability based on logistic regression with >70% for species determination.*

### Morphology, Growth, and Characteristics of Strain RW2

Strain RW2 is a Gram-positive aerobe that is facultatively anaerobic. It subsists heterotrophically with no evidence of photoheterotrophy. After 48 h of growth at 30°C on M-agar, it forms bright orange, smooth, circular colonies that are typically 3 to 4 mm in diameter (Figure 1C and Table 3). In liquid M-medium at 30°C, the cells were coccoid after 24–48 h in logarithmic growth and irregularly shaped after 72 h in stationary phase, coincident with the beginning of biofilm formation under shaking (100 rpm) (Figures 1D,E). Biofilms first appeared within ∼72 h, and their growth culminated at 8 days for 30°C under shaking (100 rpm).

**Table 3:** Morphology and growth properties under differential temperature, pH, and salinity.

	RW2	DVS3Y	DSM14480	DSM17290	DSM14481	DSM15368	JCM12280	DSM20416

Habitat of isolation	Microbialite	Marine	Microbial mat	Permafrost	Garden Pond	Glacial Water	Fish processing	Creamery
Colony size (mm)	2–4	2–3	2–3	2–3	2–4	2–4	1–5	2–5
Colony shape	Round	Round	Round	Round	Round	Round	Round	Irregular
Colony color	Orange	Orange	Orange	Orange	Orange	Yellow-orange	Orange	Yellow-orange
Growth temp (°C)								
-2.5	NA	+	+	+	+	w	w	-
4	+	+	+	+	+	+	+	-
37	+	+	+	+	+	-	+	w
40	+	-	+	+	+	-	+	-
45	+	-	-	-	-	-	-	-
50	+	-	-	-	-	-	-	-
Max pH for growth	50	30	41	40	41	30	40	37
4	-	-	-	-	-	-	-	-
5	+	-	-	-	-	-	-	-
11	+	-	-	-	-	-	-	-
Max NaCl (%) for growth								
5.80%	+	+	+	+	+	+	+	+
7.00%	+	-	-	-	-	-	-	-


*All growth temperature measurements for strain RW2 were taken after 2 days, except at 4°C which took 10 days to grow. Non-strain RW2 data are from Chaturvedi et al. (2008). no growth (-), strong growth (+), weak growth (W), data not available (NA). DVS3Y – *E. soli* DVS3Y, DSM14480 – *E. antarcticum* DSM14480, DSM17290 – *E. sibiricum* DSM17290, DSM14481 – *E. undae* DSM14481, DSM15368 – *E. indicum* DSM15368, JCM12280 – *E. oxidotolerans* JCM12280, DSM20416 – *E. acetylicum* DSM20416. All isolates are further described in publications listed in Table 1. DSM17290 – E. sibiricum DSM17290 also known as strain DSM 17290/JCM 13490/255-15 is described in Table 1.*

No flagella were observed in the electron microscopy (SEM) or under standard light microscopy (Figures 1D,E). Strain RW2 had a positive motility assay provided by API20E kit. The genome annotation of strain RW2 predicts a complete flagellum biosynthesis pathway (including the *fih, fli, fig* gene operons). Flagella staining and motility testing was positive, meaning that strain RW2 is motile with peritrichous flagella.

Strain RW2 grows at a wide range of temperatures, added salinity, and pHs. Ranging from 4 to 50°C, strain RW2 grows at the broadest range of temperatures currently reported for any isolate of *Exiguobacterium* genus) (Table 3) though we were not able to test its growth at subzero temperatures. Likewise, strain RW2 exhibits the broadest range of pH tolerance reported within the genus, from a pH of 5 to pH 11 (Table 3). We attempted to grow strain RW2 on minimal medium to test the absolute requirement of NaCl required for growth, but we were unsuccessful. We tested overall NaCl tolerance for growth by directly adding NaCl from 0 to 16% (w/vol) to M-agar/broth. Strain RW2 only grew at 0 and 7% (Table 3). Nevertheless, strain RW2 has a higher salt tolerance than other non-marine isolates, which do not grow above 7% added NaCl (Table 3). Interestingly, strain RW2 grows beyond the range of the temperature, pH, and salinity expected at 20 m depth in Pavilion Lake microbialites, where temperatures range from 4 to 10°C, and pH is very stable at 8.1–9.1 (Lim et al., 2009). This degree of abiotic tolerance makes RW2 unique among isolates of the genus.

### Genome Properties and Comparative Genome Analysis

The initial draft genome of strain RW2 was not complete and fragmented on 23 contigs (White et al., 2013a) (Table 4). With progressiveMauve, mapping, and manual finishing, we closed the strain RW2 genome into a single circular chromosome. This chromosome is 3,019,504 bp in length, with a GC content 52.05%, and no plasmids (Table 4). This is similar to the genome assembly of strain GIC31, which is likewise a single circular contig, with high coverage that suggests no evidence of plasmids (Table 4).

**Table 4:** *Exiguobacterium* genome assembly and annotation statistics.

	^∗^RW2	RW2	GIC31	N139	S17	OS-77	8-11-1	AT1b	MH3	B7	255-15
Sequencing Method	Illumina MiSeq	Illumina MiSeq	PacBio	454 GS FLX	454 GS FLX	454 GS FLX	Illumina HiSeq	454Sanger	Illumina HiSeq	SOLiD	454Sanger
Assembler	–	Ray	HGAP	NewblerMIRA	Newbler	Newbler	Velvet	PhredPhrap	SOAPdenovo	VelvetEdena	PhredPhrap
Assembly Name	–	r23K55	–	ExiN39	ExiS17_1.0	ASM41419v1	V1	ASM2304v1	ASM49663v1	ASM29943v1	ASM1990v1
Genome coverage	–	300x	–	85x	63x	45x	300x	20x	140x	> 400x	–
No. Contigs	1	23	2	23	163	23	31	1	1	1	3
Scaffolds	–	–	2	23	–	5	–	–	–	–	–
Genome Size (bp)	3019504	3019504	2974642	2952486	3127363	3151479	2906962	2999895	3164195	2815863	3040786
N50	3019504	705844	2918587	1553709	34407	349882	340222	–	–	–	–
Largest Contig (bp)	3019504	947149	2918587	1553709	122784	929170	663994	–	–	–	–
G+C%	52.1	52.1	52.1		53.1	47.1	52.8	49	47.2	47.5	47.7
Annotation Method	NCBI	NCBI	NCBI	NCBI	NCBI	RAST	RAST	NCBI	NCBI	NCBI	NCBI
Gene No.	3148	3137	3072	3108	3268	–	–	3141	3332	2941	3155
Protein coding	3092	3079	2929	2965	3218	3265	2926	3020	3203	2772	3015
Pseudogenes	0	0	44	38	–	–	–	23	41	76	–
rRNAs	10	10	28	34	2	2	2	9	9	9	9
tRNAs	46	48	67	67	48	49	14	68	60	66	69
Plasmids	0	0	1	3	–	–	–	0	0	0	2


*–, not available; Members are listed by strain name: ^∗^Draft genome published White et al. (2013a); ^#^complete genome presented here; RW2, GIC31, N139 – *E. chiriqhucha* strains; RW2, GIC31, N139 renamed in Gutiérrez-Preciado et al., 2017; S17 – *E. sp* strain S17 from Ordoñez et al. (2013); B7 – *E. antarcticum* strain B7 from Fruhling et al. (2002); OS-77 – E. sp strain OS-77 from Nonaka et al. (2014); 8-11-1 – E. sp strain 8-11-1 from Jiang et al. (2013); AT1b – E. sp strain AT1b from Vishnivetskaya et al. (2011); MH3 – E. sp strain MH3 from Tang et al. (2013); B7 – E. antarcticum strain B7 from Carneiro et al. (2012); 255-15 – E. sibiricum strain 255-15 from Rodrigues et al. (2008).*

The draft and completed genomes of *Exiguobacterium* spp. were similar regarding genome size, GC content, and the number of protein-coding genes. Within the genus, genome sizes range from 2.82 to 3.16 Mb, with GC content ranging from 47.5 to 53%, containing from 2,941 to 3,323 genes that encode 2,772 to 3,332 predicted proteins (Table 4). Strain RW2 had lower numbers of rRNAs, tRNAs, and had 17 pseudogenes predicted (Table 4). Both S17 and 8-11-1 have lower synteny, possibly as a result of the fact that these are less complete genome assemblies (>25 contigs) (Table 4 and Supplementary Figure S1). Among members of clade IIa, strain RW2 had the highest genome synteny to strain AT1b (Supplementary Figure S1). MetaCyc/BioCyc pathway predictions using Metapathways suggest that >90% of the pathways are shared even among genomes with <65% AAI (Supplementary Figure S2). Strain RW2 has the high AAI identity to strain S17 (87.76%, Supplementary Figure S2). It also shared over 50% of RAST functions with more distant relatives in the phylum *Firmicutes* (e.g., *Planococcus halocryophilus* and *Bacillus halodurans*, (Supplementary Figure S1). The rarity of unique genes in strain RW2 suggests that it has a restricted accessory genome (i.e., pan-genome) while a large core genome is highly conserved across strains of *Exiguobacterium.*

### Potential Genomic Signatures of Physiological Adaptability

Strain RW2 has physiological adaptability to a range of thermal, salinity and pHs which we further inquired the genomic basis for such growth parameters. Cold shock proteins encoded by *cspA* to *cspI* are molecular chaperones that help to regulate protein-folding under cold conditions and prevent cryodamage (Kawahara, 2008). Interestingly, strain RW2 and the other clade II members seem to lack *cspA* and *cspB* genes, while members of clade I have multiple copies of *cspA* and *cspB* (e.g., *E. sibiricum* strain 255-15 and *E. antarcticum* B7). We compared the strain RW2 genome to a distantly related member of the firmicutes known as *Planococcus halocryophilus* which can grow at -15°C (Mykytczuk et al., 2013). *Planococcus halocryophilus* has only one copy of *cspA*, unlike *E. sibiricum* strain 255-15 and *E. antarcticum* B7 which have multiple copies of *cspA*. This suggests growth below -5°C mechanisms are required.

By contrast, strain RW2 is surprisingly thermotolerant and grows at >45°C (White et al., 2013a) (Table 3). Strain RW2 genome contains a complete heat shock gene cluster (*dnaJ, dnaK*, and *GrpE*), which encodes for chaperones that prevent denaturation and aggregation of proteins at high temperatures (Feder and Hofmann, 1999).

In regards to osmoregulation, choline and betaine uptake/biosynthesis pathways are effective systems for regulating osmolyte concentrations, including that of NaCl. Uptake pathways include the genes *bet*, *opu*, and *proU*, which encode ABC transport proteins (Sleator and Hill, 2002). We again compared the strain RW2 genome to *P. halocryophilus* as the latter can tolerate 18% added NaCl (Mykytczuk et al., 2013). Strain RW2 has no *bet* genes, one copy of *opuA* (type AA, AB, AC only), no *opuC*, two copies of *opuD*, and one copy of *proU* (*proX* only); whereas *P. halocryophilus* has one copy of *betT*, has two copies of *opuA* (type AA, AB, AC), one copy *opuC*, five copies of *opuD*, multiple copies of *proU* (two copies *proV/X*, and one *proW*) based on RAST annotations.

In regards to pH regulation, strain RW2 genomic evidence of regulation includes antiporters—including the arginine-ornithine antiporter (a*rcD*)*—*can facilitate survival under acidic conditions (Fulde et al., 2014). Strain RW2 has four copies of the *arcD* antiporter—the same number found in *E. marinum* DSM16307^T^ and *E. acetylicum* DSM20416^T^, which dwell in saline marine environments. By contrast, close relatives of strain RW2, such as strain AT1b and S17 have only two copies of *arcD*; while *P. halocryophilus* has zero copies based on RAST annotation. Strains of the alkaliphilic *Bacillus* genus commonly regulate high pH with the *nhaC* Na^+^/K^+^ antiporter (Ito et al., 1997). Strain RW2 has three copies of *nhaC* gene, which is the same in AT1b (clade II) and *P. halocryophilus*, whereas *E. sibiricum* 255-15 (clade I) which only has two copies based on NCBI annotation. The *mrp* and *tet(L)* gene cluster antiporters which also regulate high pH (Padan et al., 2005), were not found in strain RW2 or other members of the genus.

### Fatty Acid Composition Under Differential Growth Conditions

**FIGURE 5:**
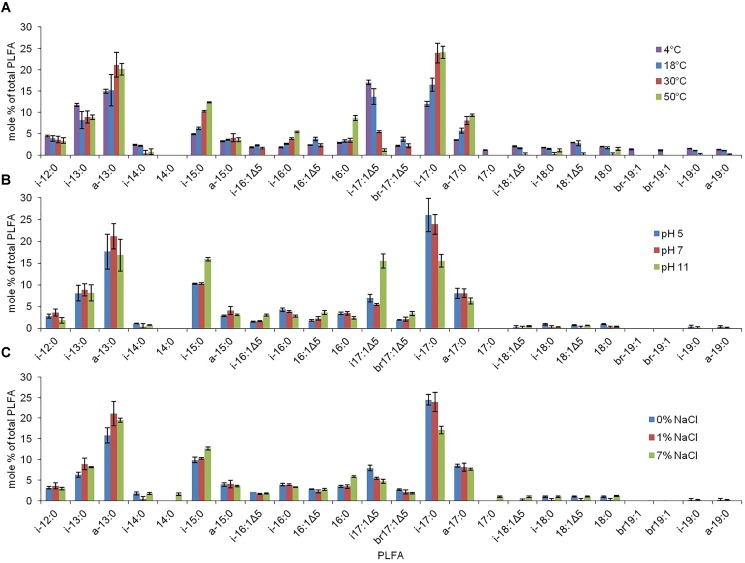
The PLFA profiles associated with strain RW2 under varying growth conditions. **(A)** Temperature. **(B)** pH. **(C)** Salinity. Growth conditions for temperature were on M-agar plates, 1% NaCl, pH 7, 1.5% agar w/v (<45°C) and 4% agar w/v (>45°C). Growth conditions for pH were on M-agar plates, 1% NaCl, 30°C, 1.5% agar w/v. Growth conditions salinity (added NaCl w/v) were on M-agar plates, pH 7, 30°C, 1.5% agar w/v. These values are % of total which are averaged from triplicate measurement with standard deviation included.

Under all growth conditions, the PLFA profiles of strain RW2 were mainly comprised of saturated branched PLFAs, predominantly anteiso-C13:0, Iso-C13:0, Iso-C15:0, Iso-C17:0, and anteiso-C17:0 (Table 5). The percentages of iso-C17:0 and anteiso-C17:0 at 23.9% and 8.1%, respectively, are the highest reported for these PLFAs for any strain of *Exiguobacterium* spp. (Table 5). In strain RW2, the fourth most abundant phospholipid is Iso-C17:1Δ^5^, at 5.5% (Table 5). This phospholipid is not found in other *Exiguobacterium* isolates (Table 5) and is consistent with strain RW2 being assigned as a new species. Iso-C17:1Δ^5^ varied by as much as 12% in response to altered growth conditions (Table 6 and Figure 5).

**Table 5:** Selected phospholipid fatty acids from strains of *Exiguobacterium.*

	StrainRW2	DSM14481^a^	DSM6208^a^	7–3^a^	K22-24^b^	DSM16307^b^
i*C*_13:0_	8.9 ± 1.4	12	18	9	12.2	1
*aC*_13:0_	21.1 ± 2.9	11	12	11	16.4	16.8
*iC*_15:0_	10.3 ± 0.2	11	4	13	14.2	14.1
*aC*_15:0_	4.0 ± 0.9	2	ND	3	5.8	2.2
*C*_16:1Δ 5_	1.6 ± 0.1	18	10	8	2.4	3.6
*iC*_16:0_	3.9 ± 0.2	ND	ND	2	3.8	6.9
*brC*_16:1_	1.6 ± 0.1	ND	ND	ND	ND	ND
*C*_16:0_	3.4 ± 0.4	13	27	17	2.8	4.7
*iC*_17:1Δ 5_	5.5 ± 0.3	ND	ND	ND	ND	ND
*iC*_17:0_	23.9 ± 2.3	5	6	9	16.1	16.8
*C*_17:0_	0	ND	ND	ND	tr	tr
*aC*_17:0_	8.1 ± 1.0	ND	ND	3	4.8	3.6
*C*_18:1Δ 5_	0.2 ± 0.3	ND	ND	ND	ND	ND
*C*_18:0_	0.2 ± 0.3	5	5	4	tr	tr
*iC*_18:0_	0.2 ± 0.4	ND	ND	ND	1.6	tr


*Values are % of total which are averaged from triplicate measurement with standard deviation included. Non-strain RW2 data are from ^*a*^Rodrigues et al. (2006) and ^*b*^Singh et al. (2013). No phospholipid detected or no data available (NA). Traces of phospholipid (<1%) detected (tr). This for standard growth conditions for strain RW2 [M-agar, 30°C, 1% NaCl (w/v) and pH 7, on 1.5% (w/v) agar]. DSM14481 – *E. antarcticum* DSM14481, DSM6208 – *E. aurantiacum* DSM6208 7-3 – *E. sibiricum* 7-3, K22-24 – *E. himgiriensis* K22-24b, DSM16307 – *E. marinum* DSM16307. All strains are further listed in Table 1.*

**Table 6:** Mole percentage distributions of PLFAs for strain RW2 grown under varying temperature, pH, and salinity conditions.

Fatty acid	4°C	18°C	30°C^∗^	50°C	pH 5	pH 7^∗^	pH 11	0% NaCl^∗^	1% NaCl^∗^	7% NaCl
*iC*_12:0_	4.4 ± 0.2	3.9 ± 0.7	3.6 ± 0.8	3.4 ± 0.7	2.8 ± 0.5	3.6 ± 0.8	1.8 ± 0.6	3.1 ± 0.3	3.6 ± 0.8	2.9 ± 0.3
*iC*_13:0_	11.8 ± 0.3	8.2 ± 2.0	8.9 ± 1.4	8.8 ± 0.5	8.0 ± 1.8	8.9 ± 1.4	8.2 ± 1.9	6.3 ± 0.6	8.9 ± 1.4	8.1 ± 0.1
*aC*_13:0_	15.0 ± 0.5	15.2 ± 3.7	21.1 ± 2.9	20.1 ± 1.4	17.6 ± 4.0	21.1 ± 2.9	16.8 ± 3.7	15.8 ± 1.8	21.1 ± 2.9	19.5 ± 0.5
*iC*_14:0_	2.4 ± 0.1	2.1 ± 0.1	0.4 ± 0.7	0.8 ± 0.7	1.1 ± 0.1	0.4 ± 0.7	0.7 ± 0.1	1.8 ± 0.4	0.4 ± 0.7	1.8 ± 0.2
*C*_14:0_	0	0	0	0	0	0	0	0	0	1.6 ± 0.3
*iC*_15:0_	4.9 ± 0.1	6.2 ± 0.3	10.3 ± 0.2	12.3 ± 0.2	10.2 ± 0.1	10.3 ± 0.2	15.9 ± 0.4	9.9 ± 0.7	10.3 ± 0.2	12.7 ± 0.4
*aC*_15:0_	3.2 ± 0.1	3.6 ± 0.1	4.0 ± 0.9	3.6 ± 0.5	2.9 ± 0.2	4.0 ± 0.9	3.1 ± 0.2	3.9 ± 0.4	4.0 ± 0.9	3.6 ± 0.2
*iC*_16:1Δ 5_	1.8 ± 0.1	2.3 ± 0.1	1.6 ± 0.1	0	1.5 ± 0.2	1.6 ± 0.1	3.0 ± 0.2	2.1 ± 0.0	1.6 ± 0.1	1.7 ± 0.1
*iC*_16:0_	1.8 ± 0.1	2.6 ± 0.1	3.9 ± 0.2	5.4 ± 0.2	4.3 ± 0.4	3.9 ± 0.2	2.8 ± 0.2	3.9 ± 0.3	3.9 ± 0.2	3.3 ± 0.1
*C*_16:1Δ 5_	2.3 ± 0.1	3.7 ± 0.4	2.3 ± 0.4	0	1.8 ± 0.2	2.3 ± 0.4	3.6 ± 0.5	2.8 ± 0.1	2.3 ± 0.4	2.7 ± 0.2
*C*_16:0_	2.9 ± 0.1	3.3 ± 0.3	3.4 ± 0.4	8.7 ± 0.6	3.4 ± 0.3	3.4 ± 0.4	2.4 ± 0.3	3.5 ± 0.2	3.4 ± 0.4	5.8 ± 0.2
*iC*_17:1Δ 5_	17.0 ± 0.5	13.7 ± 1.8	5.5 ± 0.3	1.1 ± 0.3	7.0 ± 0.9	5.5 ± 0.3	15.5 ± 1.6	7.9 ± 0.7	5.5 ± 0.3	4.7 ± 0.5
*brC*_16:1_	2.2 ± 0.1	3.6 ± 0.5	2.1 ± 0.5	0	1.9 ± 0.1	2.1 ± 0.5	3.4 ± 0.5	2.7 ± 0.2	2.1 ± 0.5	1.8 ± 0.2
*iC*_17:0_	12.0 ± 0.6	16.5 ± 1.5	23.9 ± 2.3	24.1 ± 1.5	26.1 ± 3.8	23.9 ± 2.3	15.5 ± 1.5	24.4 ± 1.3	23.9 ± 2.3	17.1 ± 1.0
*aC*_17:0_	3.5 ± 0.1	5.7 ± 0.6	8.1 ± 1.0	9.3 ± 0.3	8.0 ± 1.2	8.1 ± 1.0	6.3 ± 0.7	8.5 ± 0.4	8.1 ± 1.0	7.6 ± 0.2
*C*_17:0_	1.1 ± 0.1	0	0	0	0	0	0	0	0	1.0 ± 0.2
*iC*_18:1Δ 5_	2.0 ± 0.1	1.5 ± 0.1	0.2 ± 0.3	0	0.2 ± 0.4	0.2 ± 0.3	0.5 ± 0.1	0	0.2 ± 0.3	0.9 ± 0.2
*iC*_18:0_	1.8 ± 0.1	1.4 ± 0.2	0.2 ± 0.4	1.1 ± 0.3	0.9 ± 0.1	0.2 ± 0.4	0.3 ± 0.0	1.0 ± 0.2	0.2 ± 0.4	1.0 ± 0.2
*C*_18:1Δ 5_	2.9 ± 0.1	2.7 ± 0.6	0.2 ± 0.3	0	0.7 ± 0.1	0.2 ± 0.3	0.6 ± 0.1	1.0 ± 0.2	0.2 ± 0.3	1.0 ± 0.1
*C*_18:0_	2.0 ± 0.1	1.7 ± 0.2	0.2 ± 0.3	1.4 ± 0.4	0.9 ± 0.1	0.2 ± 0.3	0.3 ± 0.1	1.0 ± 0.2	0.2 ± 0.3	1.1 ± 0.1
*brC*_19:1_	2.4 ± 0.1	0	0	0	0	0	0	0	0	0
*iC*_19:0_	1.5 ± 0.1	1.0 ± 0.1	0.1 ± 0.2	0	0.3 ± 0.3	0.1 ± 0.2	0	0.2 ± 0.3	0.1 ± 0.2	0
*aC*_19:0_	1.3 ± 0.1	1.0 ± 0.1	0.1 ± 0.2	0	0.3 ± 0.3	0.1 ± 0.2	0	0.2 ± 0.3	0.1 ± 0.2	0
**Degree of unsaturation**	0.31	0.28	0.12	0.01	0.13	0.12	0.27	0.17	3.6 ± 0.8	0.13
**Ratio i-PLFA/a-PLFAs**	3.3	2.9	2.1	2.1	2.5	2.1	2.9	2.6	2.1	2.1
**Average chain length^∗^**	15.6	15.6	15.2	15.2	15.5	15.2	15.5	15.5	15.2	15.2


*^∗^Average chain length = [(mol % i-12:0/100)^∗^12] + [(mol % i-13:0/100)^∗^13] +…[(mol % a-19:0/100)^∗^19]*

*^∗^This for standard growth conditions for strain RW2 [M-agar, 30°C, 1% NaCl (w/v) and pH 7, on 1.5% (w/v) agar] all in triplicate. These values are % of total which are averaged from triplicate measurement with standard deviation included.*

The PLFA profiles of strain RW2 were found to undergo measurable changes over the experimental growth temperature range (4–50°C). Notably, large shifts were observed in the unique branched monoenoic PLFAs, including iso-C16:1Δ 5, iso-C17: 1Δ5, brC17:1Δ 5, iso-C18:1Δ5, and brC19:1 (Table 6 and Figure 5). The mol % of each of these PLFAs decreased by >93% between 4 and 50°C, and iso-C16:1Δ5, iso-C18:1Δ5, and brC19:1—through present at 4°C, were not detected at all at 50°C (Table 6 and Figure 5). Of all fatty acids, iso-C17:1Δ5 underwent the largest shift, decreasing from 17.0 ± 0.5 mol % at 4°C to 1.1 ± 0.3 mol % at 50°C (Table 6 and Figure 5).

Phospholipid fatty acid profile shifted across added NaCl and pH growth conditions (Table 6 and Figure 5). Similar to the temperature profiles, the notable differences were found in i-iso-C16:1Δ 5, iso-C17: 1Δ5, brC17:1Δ5, and iso-C18:1Δ5 (Table 6 and Figure 5). Under the acidic pH 5, iso-C17:1Δ5 was found to comprise 7.0 ± 0.9 mol % of the total PLFAs; this value increased to 15.5 ± 1.6 mol % by pH 11 (Table 6). This shift was the major influence on the degree of unsaturation, which increased between pH 7 and pH 11 but displayed little change between pH 5 and pH 7 (Table 6). The monoenoic PLFAs were also found to increase between pH 7 and pH 11, while there were no differences observed in these PLFAs between pH 5 and pH 7 (Table 6).

Salinity (via added NaCl) had limited effects on the composition of the PLFA profile. The total mol % of branched monoenoic PLFAs displayed a minor decrease between 0 NaCl and 1% NaCl but remained constant after that. These values decreased to 0.12 and 2.1, respectively, at 1% NaCl and remained similar at 7% NaCl (Table 6 and Figure 5). C14:0 and C17:0 were only detected at 7% NaCl (Table 6). On the contrary, the mol % of all branched PLFAs was the lowest at 7% NaCl. The average fatty acid chain length displayed no large changes with altered salinity (Table 6 and Figure 5).

The strain RW2 genome predicts the synthesis of many fatty acids, lipids, and isoprenoids. Lipids synthesis predicted by the genome include diverse phospholipids (e.g., unsaturated/saturated) and branched phospholipids, fatty acids, cardiolipin, polyhydroxybutyrates, and isoprenoids. Lipid catabolism predicted in the strain RW2 include triacylglycerols, fatty-acids, isoprenoid, and polyhydroxybutyrate.

### Resistance of Heavy Metals and Antibiotics Including Community Presence

After sequencing the metagenomes of Pavilion Lake (White et al., 2016), we questioned whether strain RW2 was part of the Pavilion Lake microbialite microbiome. With metagenomic recruitment analysis, we uncovered the presence of strain AT1b and unclassified *Exiguobacterium* spp., with the latter being significantly (*p*-value < 0.01) enriched within the microbialite, compared to the surrounding sediment and water column (Supplementary Figure S3A). We found no *Exiguobacterium* spp. reads present in the sediment metagenomes of Pavilion Lake (Supplementary Figure S3A).

This taxonomic resolution of MG-RAST was not sufficient to detect whether the hits were from strain RW2 or another *Exiguobacterium* spp. To calculate the presence and abundance of strain RW2, we recruited metagenomic reads (from the 20 m depth microbialite sample against genomic databases from strains RW2, AT1b, and S17. Strain RW2 recruited more reads (0.46% of reads) than S17 (0.33%) or AT1b (0.25%—with its genome achieving nearly complete coverage—as well as more reads with 100% identity hits (Supplementary Figure S3B). We further checked whether any of the 100–95% identity hits recruited from the metagenome were related to heavy metal resistance genes present in the genome of strain RW2, using tBLASTx (1e^-3^). We found positive heavy metal gene recruitment to mercuric reductases, arsenic resistance pumps, multidrug and toxin efflux (MATE) resistance pumps, chromate transporters and cadmium-cobalt-zinc resistance genes.

By contrast, we could not determine which taxa were responsible for contributing antibiotic resistance genes to the Pavilion Lake microbialite metagenomes, other than *Proteobacteria* (White et al., 2016), which were affiliated with *gyrB* antibiotic resistance genes, known to encode multifunctional DNA repair proteins. Nevertheless, the assembled genome of strain RW2 encodes four copies of MATE resistant pumps, vancomycin B-type resistance proteins, tetracycline resistance MFS pumps, as well as four antibiotic biosynthesis monooxygenases, which are involved in many diverse processes including the biosynthesis of antibiotics.

Little is known about antibiotic sensitivity and metabolism in *Exiguobacterium* spp. Other than *E. soli*, *E. indicum* and *E. acetylicum*, isolates of *Exiguobacterium* spp. are sensitive to clindamycin, as is strain RW2 (Supplementary Table S2). Additionally, strain RW2 was tested against eleven other antibiotics (Supplementary Table S2) and found only to possess resistance against sulfisoxazole (300 μg). The *sul* and *dhf* genes are found in organisms resistant to sulfisoxazole (Barman et al., 2010). This resistance may stem from the genomic presence of well-known sulfisoxazole resistance genes *sul* and *dhf* genes (encoding dihydropteroate synthase and dihydrofolate reductase), within the genomes of strain RW2, *E. indicum*, and *E. acetylicum.* While genes for vancomycin and tetracycline resistance were present in the genome of strain RW2, testing revealed the strain was sensitive to both antibiotics (Supplementary Table S2).

### Carbohydrate, Amino Acid, and Nitrogen Metabolism

Biochemical tests indicated that strain RW2 uses a variety of monosaccharides, polysaccharides, and amino acids as carbon sources (Table 7). Pathways for lactose uptake were present, but only a *lacZ* (β-galactosidase) for lactose utilization appeared to be present (Table 7). Strain RW2 could not use D-mannitol, D-inositol, D-sorbitol, L-rhamnose, D-melibiose, and L-arabinose as sole carbon sources (Table 7). D-mannitol utilization was predicted by the genome, yet it was not assimilated as a sole carbon source, suggesting that only fermentation occurs (Table 7). Amygdalin was utilized by strain RW2 (Table 7), as is the case for several members of the genus. Although amygdalin metabolism was not specifically predicted in our RAST analysis of the genome, many general function beta-glucosidases were present in the genome, potentially lending this function. The genome of strain RW2 also appears to possess metabolic potential to perform various forms of fermentation including mixed acid, lactate, and acetyl-CoA fermentation to butyrate. Lastly, our analysis suggests diverse substrates including chitin, *N*-acetylglucosamine, maltose, maltodextrin, trehalose, glycerol, glycerol-3-phosphate, glycogen, fructose, gluconate, ribose, fructooligosaccharides (FOS), raffinose and deoxyribose can be used, although none were tested as growth substrates here.

**Table 7:** Biochemical tests for selected strains of *Exiguobacterium.*

Activity for	RW2	DVS3Y	DSM14480	DSM17290	DSM14481	DSM15368	JCM12280	DSM20416
Catalase	+	+	+	+	+	+	+	+
Oxidase	-	+	+	+	+	+	+	+
β-Galactosidase	+	+	+	+	+	+	+	+
Arginine dihydrolase	+	+	+	+	+	+	+	+
Lysine decarboxylase	-	+	-	+	+	+	-	-
Ornithine decarboxylase	-	+	+	+	+	+	+	+
Urease	+	-	-	-	-	-	-	-
Tryptophan deaminase	-	-	+	+	-	-	+	+
Citrate utilization	+	-	-	+	-	+	+	+
Gelatinase	+	+	+	+	+	-	+	+
Production of:								
*H_2_S*	-	-	-	-	-	-	-	-
Indole	-	-	-	-	-	-	-	-
Acetoin	-	+	+	+	+	+	+	+
Assimilation of:								
D-Mannitol	-	-	-	-	+	-	+	+
Inositol	-	+	-	-	-	+	-	-
D-Sorbitol	-	+	-	+	-	+	-	-
L-Rhamnose	-	+	-	-	-	+	-	-
D-Melibiose	-	-	-	+	-	-	-	-
Amygdalin	+	+	+	+	+	-	+	-
L-Arabinose	-	+	-	+	-	-	-	-
D-Glucose	+	+	+	+	+	+	+	+
D-Sucrose	+	+	+	+	+	+	+	+


*All growth measurements for strain RW2 were taken after 24 h in triplicate. Non-strain RW2 data are from Chaturvedi et al. (2008). All are listed by strain name. All strain publications are further described in Table 1.*

Our genome analysis suggests that strain RW2—like other members of the genus—is auxotrophic for branched-chain amino acids, as biosynthetic pathways for isoleucine, leucine, and valine were predicted to be absent, while complete degradation pathways were present. Moreover, arginine dihydrolase was functional in strain RW2; whereas, tryptophan deaminase, lysine, and ornithine decarboxylases were not (Table 7). Further growth substrates should be tested to elucidate carbohydrate and amino acid utilization in strain RW2 further.

Strain RW2 genome is predicted to encode genes that generate ammonium from amino acids. An L-asparagine biosynthetic pathway is predicted to encode genes for aspartate aminotransferase, aspartate-ammonia ligase, and L-asparaginase. The enzyme L-asparaginase is known to hydrolyze L-asparagine to L-aspartate and ammonia, though we did not chemically validate its function in strain RW2. By contrast, urease—another enzyme capable of ammonification, via the catabolism of urea into ammonium and CO_2_—was not found in the genome of strain RW2. Interestingly, cells of RW2 nonetheless measured as positive on a biochemical urease test, conducted in triplicate (Table 7). It is possible that a poorly annotated gene encodes this urease function; since there are five copies of unspecified aminohydrolases within the genome of strain RW2, and given that aminohydrolases represent the enzymatic superfamily to which urease belongs (EC 3.5.1.5).

### Carotenoid Biosynthesis

**FIGURE 6:**
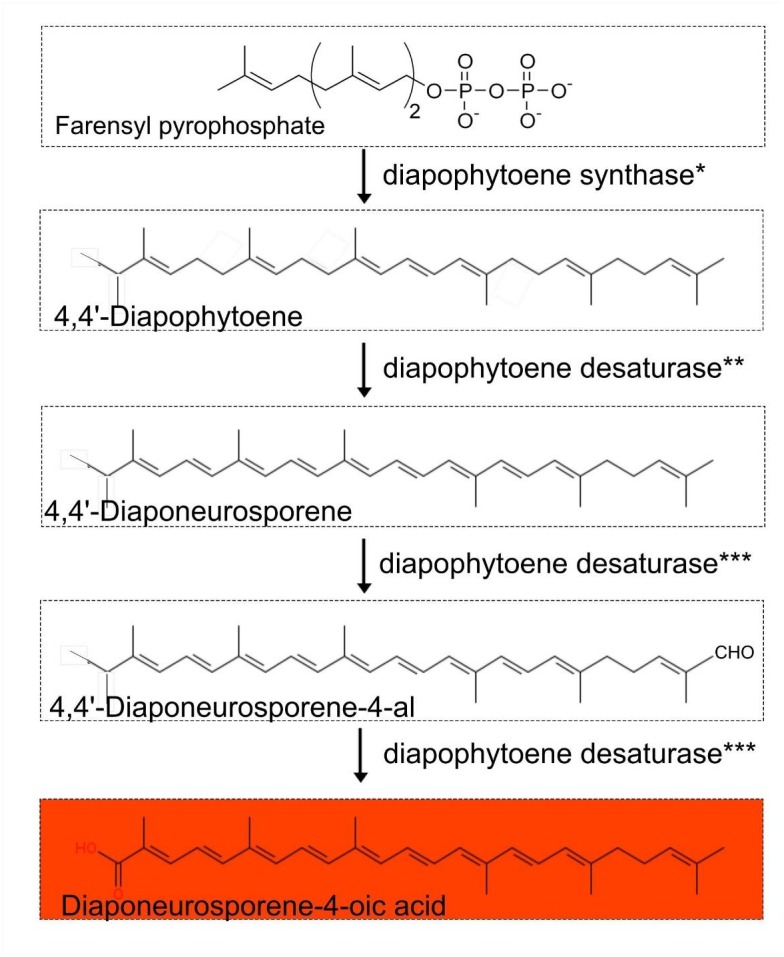
Proposed carotenoid biosynthetic pathway (4-4′ diapocarotenoids C_30_ carotenoid-based) in the genus *Exiguobacterium.*^∗^Diapophytoene synthase (*crtM*) annotated as phytoene synthase. ^∗∗^Diapophytoene desaturase (*crtN*) annotated as phytoene desaturase. ^∗∗∗^Diapophytoene desaturase (*crtNb*) annotated as phytoene desaturase.

The pathways responsible for the characteristic orange pigmentation of *Exiguobacterium* spp. are unknown, though the main candidate is that of carotenoid biosynthesis, as this pathway is commonly found in members of the *Firmicutes* (Klassen, 2010). Our comparative genomic analysis indicates that complete pathways for carotenoid C_30_ biosynthesis were present in all the published *Exiguobacterium* spp. genomes, including that of strain RW2 (Figure 6). Relevant enzymes include diapophytoene synthase (*crtM*, annotated as phytoene synthase), diapophytoene desaturase (*crtN*, annotated as phytoene desaturase), and diapophytoene desaturase (*crtNb*, annotated as the second copy of phytoene desaturase). Similar to the case in *Halobacillus halophilus* (Köcher et al., 2009) (Figure 6), all genes for the pathway are located on a single operon, specifically on a span of 5,673 bp, stretching from position 39,293 to 44,966 bp on the genome.

## Discussion

### Exiguobacterium Coloration Is Based on C_30_ Carotenoids With Potential Functions

Based on our comparative genomic analysis of draft and completed genomes from NCBI, we predict that a 4-4’ diapocarotenoids C_30_ carotenoid-based biosynthetic pathway is responsible for the orange pigmentation of *Exiguobacterium* colonies, with diaponeurosporene-4-oic as the final product. As reported in *Halobacillus halophilus*, genes encoding this pathway are arranged as an operon, and genetic ablation of the terminal gene (for diapophytoene desaturase) results in the accumulation of diaponeurosporene-4-oic acid and consequent orange pigmentation (Köcher et al., 2009).

To our knowledge, a hypothesis has not been advanced to explain the function of pigments in the genus *Exiguobacterium.* Low concentrations of dissolved organic carbon (DOC) in Pavilion Lake allow for high UV penetration, and carotenoids might provide a “sunscreen” function to cope with these conditions (Lim et al., 2009; Lionard et al., 2012). Three tested strains of *Exiguobacterium* are known to use photoenzymatic repair (PER) and nucleotide excision repair (NER) to recover from UV-B photodamage, but all had some form of UV-B resistance beyond PER and NER (Gutiérrez-Preciado et al., 2017). We hypothesize that 4-4’ diapocarotenoids C_30_ carotenoids protect members of *Exiguobacterium* from UV-B damage, given the well-known role of carotenoids quenching and scavenging reactive oxygen species, releasing excessive light energy, and sunscreening (Wada et al., 2013). Further experimentation is needed to confirm if carotenoids indeed protect firmicutes against UV radiation.

### Potential Contribution to Microbialite Formation and Maintenance

While strain RW2 has many of the physiological features of an environmental generalist, it was specifically enriched in the microbialite community at 20 m depth in Pavilion Lake. Specifically, its reads were absent in the sediment metagenome sampled nearby and are poorly represented in the metagenome sampled from the water column overhead. Moreover, reads from strain RW2 comprised a higher proportion of total microbialite reads than those of strain S17 in Socompa Lake microbialites and strain AT1b in a hyperthermal microbial mat (Ordoñez et al., 2013, 2015). Together these data suggest that strain RW2 is a numerically significant component of the Pavilion Lake microbialites. Strain RW2 was found in lower amounts on the filters (i.e., water column metagenomes), and wasn’t as prevalent as in the microbialite metagenomes. Filters can contain small amounts of microbialite microbial mat as they were sampled near the microbialites at depth; however, the water column metagenomes are distinctly different via PCA analysis from the microbialite metagenome (White et al., 2016).

Since we do not know how long strain RW2 has lived on freshwater microbialites, it is unclear how and to what extent it has co-evolved with this microbial community. Even in the absence of evolutionary specialization, there are several ways in which strain RW2 might contribute to microbialite formation and maintenance. For instance, biofilm formation has previously been nominated as a survival mechanism in nutrient-poor conditions (Shao et al., 2013), and this might apply to the cold, oligotrophic conditions from which strain RW2 was isolated (Lim et al., 2009). By adhering to sediment and precipitated carbonates, Firmicutes such as *Exiguobacterium* could, in turn, contribute to the coherence and spatial structure of microbialites like those found in Pavilion Lake and Laguna Negra (White et al., 2016; Gomez-Javier et al., 2018). But biofilm formation is not necessarily a consequence of co-evolution with microbialites since other strains form biofilms in the absence of microbialites, such as *Exiguobacterium* strain GIC31, which may grow directly on glacial ice. A few strains of *Exiguobacterium* isolates have been found to be endemic to Cuatro Ciénegas Basin which microbialites are found (Rebollar et al., 2012).

Several metabolic pathways predicted in strain RW2 might also contribute to microbialite formation by promoting inducing carbonate precipitation via urease or deamination of amino acids. For instance, the enzyme L-asparaginase generates ammonium as a by-product of the deamination of amino acids, thereby increasing alkalinity, which favors the precipitation of carbonate minerals (Castanier et al., 1999). Interestingly, our biochemical assay indicated that urease is also active in strain RW2; though we found no evidence of urease genes within its genome, nor that of any other *Exiguobacterium* strain. This suggests either (A) the presence of an uncharacterized “orphan enzyme” (perhaps any of the five amidohydrolases predicted in the genome of strain RW2), or (B) that a known enzyme is capable of an undescribed side reaction (Sorokina et al., 2014; Montgomery et al., 2015). Amidohydrolases belong to the same protein superfamily as urease; which is a future target for further characterization and investigation. In any case, urease—like asparaginase—can induce calcium carbonate precipitation, and it is found in carbonate precipitating members of *Bacillus* spp. (Hammes et al., 2003), though its actual involvement in biomineralization remains to be demonstrated.

More compelling is the possibility that strain RW2 contributes other metabolic functions—namely heavy-metal metabolism—since these *Exiguobacterium* genes are highly represented in the microbialite metagenome of Pavilion Lake. For reasons that are still unclear, heavy-metal metabolism appears to be an accessory feature of microbialite communities (Ruvindy et al., 2016; White et al., 2016; Kurth et al., 2017; Gomez-Javier et al., 2018). Generally, heavy metals (such as arsenic) limit microbial growth. However, we find three examples of thriving modern microbialite ecosystems in the presence of high arsenic; the microbialites of Laguna Brava (Sancho-Tomás et al., 2018), Socompa Lake (Kurth et al., 2017), and Laguna Negra (Gomez-Javier et al., 2018). The extracellular polymeric substances (EPS) within cyanobacterial microbialite mats and biofilms bind, concentrate, and remove heavy metals from the water column (Arp et al., 1999). Any heterotroph that eliminates and detoxifies heavy metals as a by-product of its metabolism would benefit the entire microbialite microbiome and would presumably be rewarded with more substrates for growth. Strangely, Pavilion Lake has low levels of zinc (0.01–0.03 mg L^-1^) and undetectable levels of cobalt, copper, chromium, arsenic, and cadmium (Lim et al., 2009).

Genes predicted in the resistance of mercury, arsenic, chromate, cadmium, cobalt, zinc resistance were all found in Pavilion Lake microbialite microbiome based on recruitment of their metagenomic reads to the genome of strain RW2. While strain RW2 hasn’t been tested for arsenic resistance; strain N139 can grow at 100 mM of arsenate (As [V]), and 2.5 mM of arsenite (As [III]) (Ordoñez et al., 2015). All three strains (RW2, GIC31, N139) have no *acr3* based on genome predictions; whereas S17 does and can grow at 10 mM As [II] (Ordoñez et al., 2015). We would predict that strain RW2 would have similar arsenic resistance as strain N139, high As [V] resistance but low As [III] due to the lack of the *acr3* gene.

Moreover, the genome of *Agrococcus pavilionensis* strain RW1—which was isolated from the same microbialite as strain RW2—also carried genes for heavy-metal resistance genes (White et al., 2013a). One possible explanation is that these genomic signatures are relicts from a time when Pavilion Lake had higher levels of dissolved heavy metals, as likely occurred in the early 20th century when mining operations were active in the region (Stevenson, 1940).

Another possibility is that metal resistance genes have other secondary alternative functions, as in *Rhodobacter sphaeroides* is known to have arsenic resistance genes expressed under high salt stress (Tsuzuki et al., 2011). For instance, the heavy metal resistance proteins genes in strain RW2 are retained in a low heavy metal environment because of the act as can also detoxification of other substrates (e.g., antibiotics). As such, heavy metal metabolism can drive co-selection of antibiotic resistance when aquatic systems that are impacted by agriculture or antibiotics introduced through wastewater and agricultural runoff anthropomorphic means (Seiler and Berendonk, 2012). Resistance in heavy metals has conferred resistance to antibiotics in a complex microbiome (e.g., chicken guts) (Nisanian et al., 2014). It is not clear whether this is the case for microbialites in Pavilion Lake, though strain RW2 also contributes antibiotic resistance genes to the metagenome of these microbialites, including those for sulfisoxazole resistance (White et al., 2016).

### Classification of the Strains as the Same Species

Using 16S rRNA gene sequence phylogeny, whole-genome ANI/AAI, and digital DNA–DNA hybridization; we confirmed that strain RW2, GIC31, and N139 belong to same bacterial species—the recently described *Exiguobacterium chiriqhucha* (Gutiérrez-Preciado et al., 2017). We also provide the first complete genomes for this species—represented by our assembly of strain RW2, as well as our the complete genome of strain GIC31. Each genome consists of a single circular chromosome, with strain GIC31 also possessing one plasmid.

### Adaptation to a Wide Range of Abiotic Conditions

While strain RW2 has a modest tolerance for added salts, it exhibits both the highest thermal growth range (4–50°C) and broadest pH growth range (5–11 pH) ever reported for the genus *Exiguobacterium.* Strain RW2 genome encodes molecular chaperons (*csp* and *dnaK*), ABC transporters, antiporters, for thermal, freezing and osmotic stress adaptation. This is unusual, a few of these extremes would be expected in the permanently cold and alkaline habitat from which this isolate was obtained. Growth at such high temperatures and low salinities is not a requirement for life in Pavilion Lake. Though there are numerous hot springs in southwestern British Columbia (Lim et al., 2009), and geothermally heated microhabitats have been known to provide refugia for certain flora during glacial periods Alternately, if strain RW2 is a recent colonist of microbialite habitats, then these physiological tolerances might be holdovers from a more generalist lifestyle. But speculation aside, in the present environment of our isolate, cold and alkaline conditions are the most likely sources of abiotic stress.

Cold stress generally decreases membrane fluidity and causes ice crystal formation, leading to cryodamage of the cell (Ponder et al., 2005). At lower temperatures (4°C), strain RW2 synthesizes a higher proportion of branched, unsaturated fatty acids (i.e., iso-C16:1Δ5, iso-C18:1Δ5, and brC19:1), which likely help prevent membrane crystallization. This increase in unsaturated fatty acids is similar to that found in *P. halocryophilus* when grown at sub-zero temperatures (Mykytczuk et al., 2013).

Its thermotolerance is unique since, until now, *Exiguobacterium* spp., strains that grow at >45°C have only been isolated from such hyperthermal environments (Crapart et al., 2007). Conversely, the percentage of unsaturated fatty acids decreased at higher temperatures. Indeed, the largest change in the PLFA composition of strain RW2 was prompted by increasing the temperature to 50°C, as branched monoenoic (unsaturated) PLFAs decreased as much as 93% between 4 and 50°C. This changing membrane profile is a characteristic cellular response to heat stress, as high levels of saturated fats decrease membrane fluidity and thereby prevent phase transitions at high temperature (Hazel, 1995). As with its lipid profile, the genome of strain RW2 seems to reflect its broad thermal and pH tolerance. It possesses a full heat-shock protein cascade (i.e., *dnaJ, dnaK*, and *GrpE)*, as does its hot-spring dwelling relative, strain AT1b.

Strain RW2 considerable tolerance of acidity is surprising, as similar acidic pH tolerance has only been reported from marine isolates of *Exiguobacterium* (i.e., *E. profundum, E. aestuarii*, and *E. marinum*) which can grow at a pH of 5.5 (but not the upper limit of pH 11 as in strain RW2) (Kim et al., 2005; Crapart et al., 2007). Our genomic annotations indicate that strain RW2 possesses the antiporter encoded by *arcD* (Fulde et al., 2014), and a Na^+^/K^+^ antiporter encoded by *nhaC*, which—in other members of *Exiguobacterium*—are known to regulate responses to acidity and alkalinity, respectively (Ito et al., 1997). Under decreasing pH, PLFAs increased in saturation, presumably resulting in a more ordered and rigid membrane. Such increases in saturation are thought to help resist H^+^ influxes under acidic conditions (Mykytczuk et al., 2013). Compared to its responses to pH and temperature, PLFA composition in strain RW2 was less affected by salinity, though the percent of saturated PLFAs increased slightly at low salinities as would be expected to increase membrane rigidity and reduce the risk of lysis (Ponder et al., 2005). It is also conceivable that strain RW2 could adapt to high pH via cardiolipin biosynthesis since alkaliphilic strains of *Bacillus* are known to upregulate this pathway under high pH (Clejan et al., 1986). Further experimental evidence is required to understand how strain RW2 regulates its responses to changes in pH.

Strain RW2 can grow from 0 to 7% added NaCl, and could potentially respond to regulate low salt stress higher osmotic stress by increasing intracellular NaCl concentration using via the choline and betaine uptake and biosynthesis pathways encoded in its genome. Halotolerance of strain RW2 is considerably lower than marine isolates found in the clade IIa, such as *Exiguobacterium aestuarii*, which can grow at 17% added NaCl (Kim et al., 2005). Genome prediction suggest strain RW2 potentially regulates NaCl stress via choline and betaine uptake and biosynthesis pathway. ABC transporter genes required for higher salinity growth were present in the genome of strain RW2 (i.e., *opuC, proW*). However, strain RW2 was missing the *bet* genes thought to be required for high salinity growth (>10%) (Mykytczuk et al., 2013). *P. halocryophilus* has *bet* genes required for high salinity growth and can grow in 18% added NaCl (Mykytczuk et al., 2013). The heat shock *dnaK* gene cluster also can also protect cells from hyperosmotic shock induced by salt (i.e., NaCl/KCl) and carbon substrates (e.g., sugars) (Bianchi and Baneyx, 1999). Overall, the temperature, pH, and salinity-based changes in its PLFA composition seem to reflect strain RW2 adaptability likely due to membrane restructuring.

## Conclusion

*Exiguobacterium chiriqhucha* strain RW2 was isolated from microbialite cyanobacterial mat within a Pavilion Lake in southeastern BC. Currently, strain RW2 has the widest pH and temperature growth range for any isolate of *Exiguobacterium.* We described the physiological, genomic, and fatty acid composition under different growth conditions for strain RW2 widely adaptability to various temperatures, pHs, and salinities. We revealed the putative pathway for the orange colony formation in *Exiguobacterium* as 4-4’ diapocarotenoids C_30_ carotenoid-based biosynthetic pathway using comparative genomic analysis. We suggest the potential role of these carotenoid pigments as protective potentially against photodamage or act as quenchers for reactive oxygen.

Our results suggest that strain RW2 is present within microbialite microbiome of Pavilion Lake. Metagenomic recruitment suggest genes related to the detoxification of heavy metals and antibiotics are supplied by strain RW2 genome. Strain RW2 is a robust biofilm former, and putatively increases alkalinity leading to carbonate precipitation is deamination of amino acids to ammonia (i.e., L-asparaginase/urease).

The remarkable range of possible growth conditions and metabolic potential of strain RW2 may make this species relevant to industries that are exploring the use of *Exiguobacterium* spp. For example, its predicted heavy-metal tolerance makes it an excellent candidate for bioremediation applications (Park and Chon, 2016), and its ability to produce carotenoids may have applications in natural pigment biosynthesis. Strain RW2 is also highly amenable to cultivation because it can grow on a diverse set of nutrients. Lastly, the ability to catabolize urea despite the apparent lack of genes encoding urease may lead to the discovery of a hitherto unknown protein or enzymatic pathway that could function in the transformation of nitrogen.

## Author Contributions

RW designed the study, and collected and plated the isolate, performed growth studies, extracted DNA, prepared libraries, assembled and annotated the genome, performed comparative genomic and phylogenetic analysis. SS completed PLFA, with financial support from GS. EG performed culturing experiments. RW preserved cells for scanning electron microscopy, which were imaged by GG. RW and GG wrote the manuscript. All authors participated in the manuscript drafting process.

## Conflict of Interest Statement

The authors declare that the research was conducted in the absence of any commercial or financial relationships that could be construed as a potential conflict of interest.

## Footnotes

1 http://stothard.afns.ualberta.ca/cgview_server/

2 http://lycofs01.lycoming.edu/~newman/AAI/html

3 http://enve-omics.ce.gatech.edu/

## Supplementary Material

Figure S1 Genome synteny and functional Venn diagrams (SEED) with Amino acid identity score (AAIr) to strain RW2. Genomes are listed in megabase pairs (Mb). Red dots are positive blast hits based on the RAST genome comparison module. Amino acid identity (AAIr) was calculated using the RAST functional module with the web-based tool (Krebs et al., 2013). Venn diagrams are listed by strain name based on RAST functional annotations (SEED/FigFams).

Figure S2 MetaCyc pathway annotations of genomes for isolates of *Exiguobacterium.* **(A)** Venn diagram high amino acid identity score (AAIr) to strain RW2 (>80% AAIr), within clade II. **(B)** Venn diagram low amino acid identity score (AAIr) to strain RW2 (<62% AAIr), within clade I. Labels on Venn diagrams, are listed by strain name with *Exiguobacterium* sp. for strains 8-11-1, AT1b, MH3, RW2, S17, followed *E. sibiricum* strain 255-15 and *E. antarcticum* strain B7.

Figure S3 *Exiguobacterium* presence in Pavilion Lake microbialite metagenome. **(A)** *Exiguobacterium* STAMP post-havoc confidence interval plots (>95%) based on ANOVA parameters for novel metabolic potential differences for microbialites and surrounding environment (using multiple groups). **(B)** Metagenomic recruitment plot of Pavilion Lake 20 m microbialite reads (∼7.5 million) to three *Exiguobacterium* genetically related to strain RW2 isolated from 20 m microbialites (minimum identity > 70% and an E-value > 1e^-5^).

Table S1 Complete genomes of *Exiguobacterium* published (as of July 2018).

Table S2 Antibiotic susceptibility for selected strains *Exiguobacterium.* Resistant (+), sensitive (-), data not available (NA). Additional data are from ^1^Chaturvedi et al. (2008). All growth measurements for strain RW2 were taken after 24 h. This for standard growth conditions for strain RW2 [M-agar, 30°C, 1% NaCl (w/v) and pH 7, on 1.5% (w/v) agar] all in triplicate.
