# Supplementary figures and images for: The Complete Genome and Physiological Analysis of the Eurythermal Firmicute Exiguobacterium chiriqhucha Strain RW2 Isolated From a Freshwater Microbialite, Widely Adaptable to Broad Thermal, pH, and Salinity Ranges
Source: Front Microbiol. 2019 Jan 8;9:3189. doi: 10.3389/fmicb.2018.03189 (PMC6331483; doi:10.3389/fmicb.2018.03189)

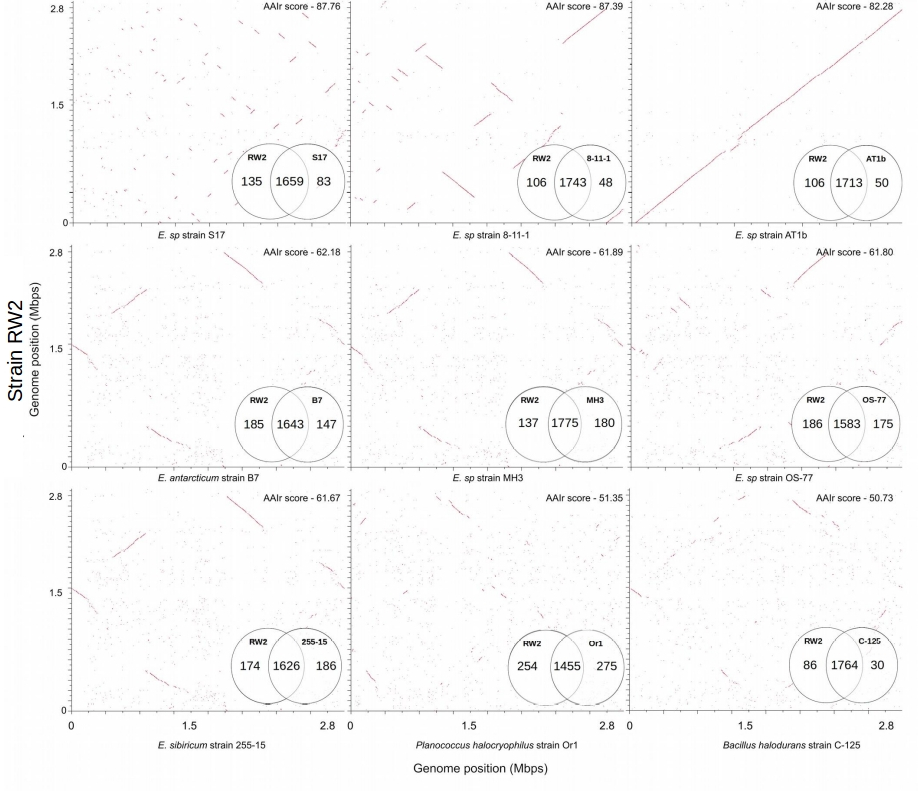

Supplement: Figure S1 — Genome synteny and functional Venn diagrams (SEED) with Amino acid identity score (AAIr) to strain RW2. Genomes are listed in megabase pairs (Mb). Red dots are positive blast hits based on the RAST genome comparison module. Amino acid identity (AAIr) was calculated using the RAST functional module with the web-based tool (Krebs et al., 2013). Venn diagrams are listed by strain name based on RAST functional annotations (SEED/FigFams). [file Image_1.JPEG]

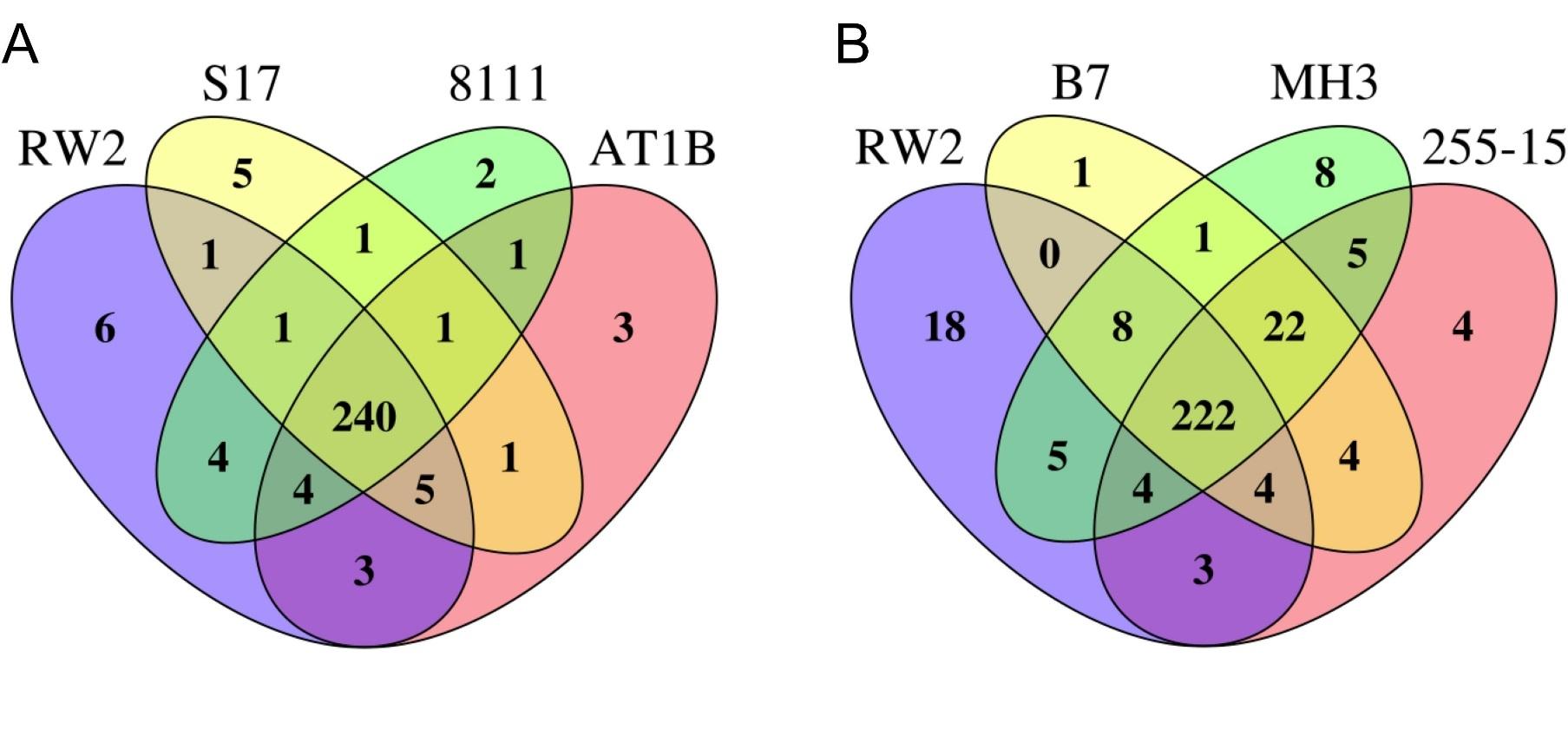

Supplement: Figure S2 — MetaCyc pathway annotations of genomes for isolates of Exiguobacterium. (A) Venn diagram high amino acid identity score (AAIr) to strain RW2 (>80% AAIr), within clade II. (B) Venn diagram low amino acid identity score (AAIr) to strain RW2 (<62% AAIr), within clade I. Labels on Venn diagrams, are listed by strain name with Exiguobacterium sp. for strains 8-11-1, AT1b, MH3, RW2, S17, followed E. sibiricum strain 255-15 and E. antarcticum strain B7. [file Image_2.JPEG]

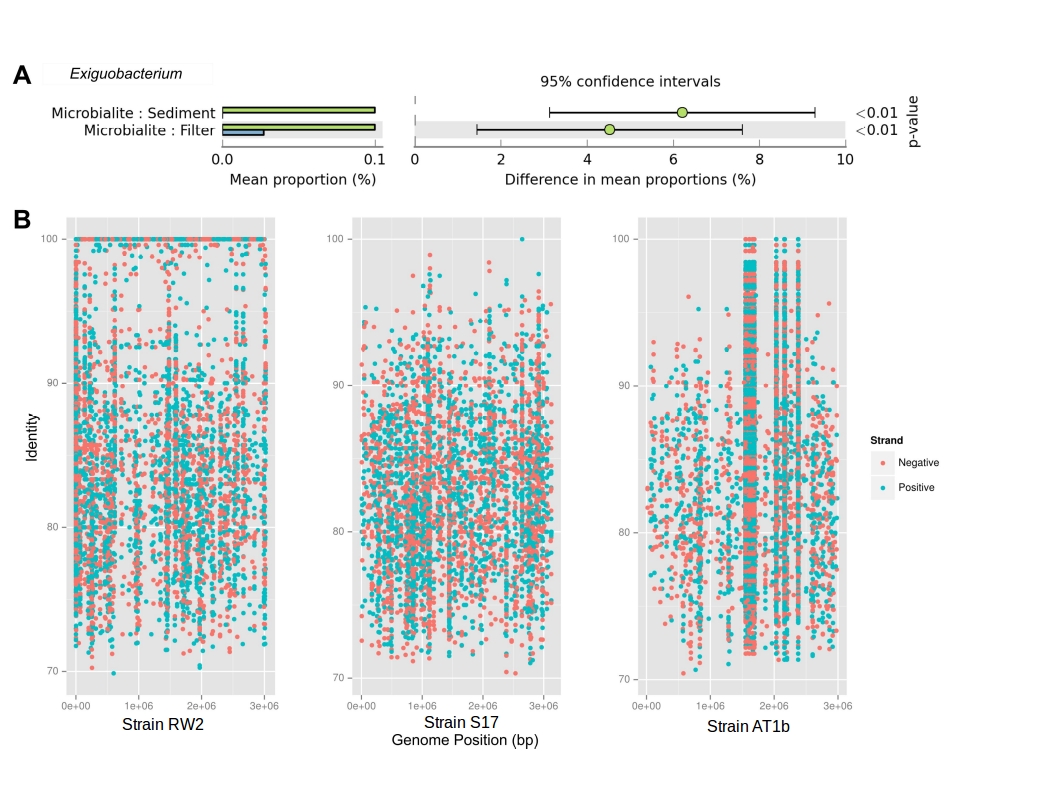

Supplement: Figure S3 — Exiguobacterium presence in Pavilion Lake microbialite metagenome. (A) Exiguobacterium STAMP post-havoc confidence interval plots (>95%) based on ANOVA parameters for novel metabolic potential differences for microbialites and surrounding environment (using multiple groups). (B) Metagenomic recruitment plot of Pavilion Lake 20 m microbialite reads (∼7.5 million) to three Exiguobacterium genetically related to strain RW2 isolated from 20 m microbialites (minimum identity > 70% and an E-value > 1e-5). [file Image_3.JPEG]
